# The “Archeology of the Light”:  A multiproxy, interdisciplinary and experimental approach to Paleolithic subterranean activities.

**DOI:** 10.12688/openreseurope.17712.1

**Published:** 2024-10-14

**Authors:** Mª Ángeles Medina-Alcaide

**Affiliations:** 1 PACEA-UMR CNRS 5199, Bâtiment B2 Allée Geoffroy Saint Hilaire, Universite de Bordeaux, Pessac CEDEX, 3615, France; 2 Universidad de Córdoba, UCO-Prehistoria, HUM-781, Córdoba, 14071, Spain

**Keywords:** Lychnology, Pyroarchaeology, Paleolithic Cave, Paleolithic Art, Internal Archaeological Context, charcoal, soot.

## Abstract

**Background:**

The "Archeology of the Light" (A-Light) project aims to improve our knowledge of paleolithic cave activities through an interdisciplinary methodology applied to rarely-studied remains: the residues of Paleolithic light from lamps, fireplaces and torches (specially, charcoal and soot).

**Methods:**

The methodology includes different stages such as: 1. Work in caves and sampling, 2 Laboratory analyses (multi-analytical approach adapted to the type of combustion residue analysed, including Anthracology, C14 dating, Bayesian analysis, SEM-EDX, TEM.EDX, Raman…), 3. Ethnographic review of firelight, 4. Experimental reproduction and monitoring of Palaeolithic firelight.

**Results:**

This approach contributes multifaceted data about the Paleolithic activities inside the caves (lighting systems selected, fuel used, chronology and intensity of visits, paleo-paths...). Besides, experimental reproductions have enabled evaluation of their lighting potential, and provide essential information for research the visibility and the accessibility of Rock Art from GIS, and allow to more realistic virtual simulation.

**Conclusions:**

In short, these data demonstrate that the *Archaeology of the Light* is here to stay and that it is an essential approach for a holistic understanding of Palaeolithic caves.

## Plain language summary

Our ancestors visited the deep areas of caves at least 176,000 years ago, although most of the archaeological remains date back 40,000 years, and they are related to *Homo sapiens* and the creating or visiting of cave art. In the A-LIGHT project, we are studying what our ancestors did inside caves and why they accessed them, even if it was not necessary for their survival. Specifically, thanks to various archaeometry and experimental analyses of combustion residues (charcoal and soot; remains scarcely studied at these sites so far) found inside 6 European caves and linked to Palaeolithic lighting methods. We have obtained relevant data on what artificial light resources they used to overcome the darkness of these spaces, how their torches or lamps worked, what the light they emitted was like, how long they lasted, what fuel they preferred for their manufacture, when and how often they visited these places, which areas were the most visited, etc. In summary, the study of carbonized remains inside caves is essential to understand our ancestors' exploration of the subterranean landscape and their ability to adapt to this inhospitable environment.

## Introduction

The first solid data of “palaeospeleology”, that is, the presence of ancient humans in deep caves, were related to Neanderthals. They were discovered in Bruniquel Cave (France) and dated 176 ka BP. They comprised six circular anthropic structures, made up of approximately 400 fractured speleothems, with more than 18 fire traces inside, 336 m. from the entrance ( Jaubert *et al*., 2016a). In the Upper Palaeolithic, combustion residues related to lighting proliferated in the inner recesses of caves, especially associated with graphic activity. However, archaeological research traditionally focused only on Palaeolithic Art. Analysis of the Internal Archaeological Context (i.e., including the traces of lighting) began in the second half of the 20th century ( Clottes, 1993; Clottes & Simonnet, 1990). This subject is now receiving increasing attention and is beginning to be addressed in a holistic and interdisciplinary way in order to obtain global knowledge of the palaeolithic anthropization of caves ( Arias & Ontañón, 2020; Garate, 2017; Jaubert *et al*., 2016b; Medina *et al*., 2018; Pastoors *et al*., 2017).

The main references on palaeolithic lighting were published during the 1960s–1980s ( Allain, 1965; Beaune, 1987; Delluc & Delluc, 1979). These studies focused mainly on a single type of lighting resource: mobile lamps ignited with animal fat. Remarkably, this light-tool is the least frequent in the archaeological record and the one with the least light capacity. Furthermore, these early studies were conducted at a time when physico-chemical analyses were only incipient within Archaeology. Now, there is an ideal research context to advance on this subject, using portable and non-invasive tools ( Carrasco *et al*., 2016; Donais & Vandenabeele, 2021; Hernanz, 2015; Ziemann & Madariaga, 2021).

Charcoals, related to the use of torches and firelight, are the most recurrent archaeological remains of light in the Internal Archaeological Context. Little attention has been paid to them, however, except in a few caves with exceptional conservation conditions ( Théry-Parisot & Thiébault, 2005; Théry-Parisot *et al*., 2018). In my Ph.D., I studied mainly these lighting residues and the results showed their potential for the integral understanding of palaeolithic underground anthropization, including in non-intact caves ( Medina, 2019). In this article, I present the postdoctoral project *The* “ *Archeology of Light”: a multiproxy, interdisciplinary and experimental approach to Paleolithic subterranean activities* directed by M.A. Medina-Alcaide and supervised by C. Ferrier at the PACEA-UMR 5199 Laboratory (Université de Bordeaux). This project was funded by the Fyssen Foundation with an allocation grant (call 2021). At present, the project is still ongoing in the framework of a postdoctoral contract MSCA-PF (2022–2024). The following sections present the objectives, methodology and results obtained during the first step of project.

## Methods

**Figure 1.**
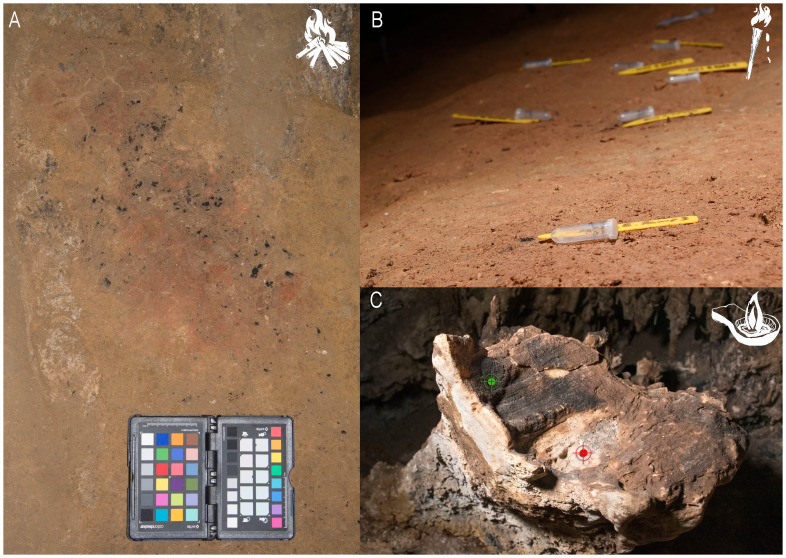
Diverse residues of Palaeolithic light systems analyzed in this project.

**Figure 2.**
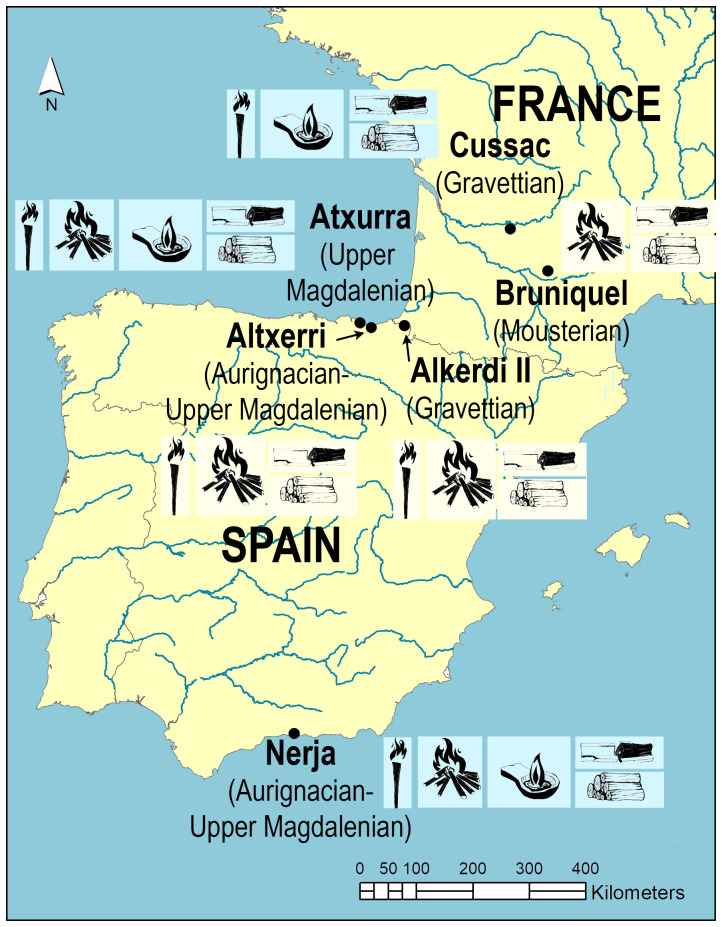
Site location, chronology, types of lighting and fuel.

In the framework of the recently published book “Light in Archaeology” ( Papadopoulos & Moyes, 2021), the main aim of this project was to improve our knowledge of the early subterranean behaviour of Humanity, through a pioneering and interdisciplinary methodology applied on seldom studied remains: **the residues of Palaeolithic light, mainly charcoals and soot from fireplaces** ( Figure 1a: *fireplace from Atxurra with remains of charcoals, ash and rubefacted clay* – Garate *et al*., 2023), **torches** ( Figure 1b: *scattered charcoals linked to the use of wooden torches from Alkerdi 2* cave) and **lamps** ( Figure 1c: *fixed “lamp” of Nerja cave with soot deposit -green- and micro-charcoal remains -red*). This research focuses on outstanding remains of palaeolithic lighting from **six European caves** ^1^, one of which has been declared a World Heritage Site by UNESCO (Altxerri cave) ( Figure 2). These remains were carefully selected in order to: (1) analyze and compare the different lighting systems, (2) extend the analysis to combustion remains of various kinds (beyond charcoals), (3) expand the archaeological record studied at the international level and (4) extend the period studied, whilst incorporating the knowledge of lighting technology possessed by different humanoid species (Neanderthal and *H. sapiens*). Specifically, the inner anthropization of the selected sites integrates different phases of the Upper Palaeolithic (35–12 ka. BP), including a period of the Middle Palaeolithic (176 ka. BP). This is conducive to a good diachronic understanding of the topic.

The **objectives** of the project are as follows: 1. Define and analyze the lighting systems used by palaeolithic hunter-gatherers in each cave, by multiproxy and multianalytical analysis (prioritizing on-site and non-destructive analysis). 2. Quantify the physical parameters and elemental characteristics, including their residual aspects, through experimental replication and monitoring. 3. Synthesize the data and evaluate the role of firelight in palaeolithic hunter-gatherer societies, including its significance for the anthropization of caves and the performance of the first symbolic activities in them.

At the **methodological level**, the A-LIGHT project comprises six Work Packages:

*1. Work in caves and sampling*: within the framework of the research projects in each cave, a systematic surface survey of each cave was carried out, as well as small probes in particular areas to assess the remains of anthropic activity underground beyond the surface level ( Medina-Alcaide *et al*., 2018). Dino-lite® portable microscopes (models AM4815ZT 20–200x, AM4013MZT4 400–470x, AM4515T8 700–900x were used to conduct a preliminary characterization of the vestiges in caves, and Disto Leica (model X2 modified) for the topographical geolocalization on the 3D or the 2D topography of the caves ( Intxaurbe *et al*., 2020; Trimmis, 2018). The intrinsic and extrinsic characteristics of each combustion residue were recorded for the study. Sampling was carried out as aseptically as possible so as not to contaminate the sample for dating purposes, and was done either manually for the remains on the surface or using a flotation system for the remains from probes.

*2. Laboratory analyses*: On the charcoals, holistic anthracological analysis was implemented, covering, if possible, a taxonomic approach (to characterize the woody species of origin), a taphonomic approach (to identify the physiological and phenological state of the wood) and a dendro-anthracological approach (to determine the thickness of the wood). Raman analysis was carried out on some of the charcoals to determine the maximum combustion temperature, following Deldicque *et al*. ( 2016; 2023). Lastly, 14C-AMS dating of the charcoals was used to determine the date of human use of each cave.

The project also includes the use of various laboratory tools for the determination and study of lighting remains: binocular loupe, light microscope, SEM-EDX and TEM-EDX. The latter two tools include the Energy Dispersive X-ray spectroscopy technique which enables the analytical profiles and distribution maps to be obtained using the dispersed energy. This helps to characterize the nature of the remains that do not present an identifiable structure. Raman and TEM-EDX spectroscopy proved extremely useful to characterize the prehistoric soot remains. This project also included collaboration with other specialists and laboratories, for example, for the fuliginochronological analysis (S. Vandevelde, Université du Québec), for Raman analysis (D. Deldicque, École Normale Supérieure), for GC-AMS analysis and determination of the fatty residues (F. Lafont, University of Cordoba) or for micromorphology analysis, to identify the ash and rubefaction clay, and various phases of visits inside the caves (C. Ferrier, Université de Bordeaux and M. Arriolabengoa, University of the Basque Country).

3. Ethnographical review of firelight: This project includes an exhaustive review of the ethnographic literature on firelight in circumpolar societies (Evenks, Inuits), frequently compared in the literature with Palaeolithic groups for their lifestyles, and on the last human groups to live in caves (the Tao't Bato tribe). Altogether, this will allow the acquisition of techniques and knowledge (assembly system, operation ...) on traditional fire lighting systems that will be fundamental for the development of the experimental activity. This information is key to exploring the social and cultural meaning of firelight beyond practical activity. For example, there is a curious cultural relationship between the qulliq (fat lamp) and Inuit women ( Billson & Mancini, 2007).

4. Reproduction and monitorization of Paleolithic light: The experimental replication in a subterranean context under controlled conditions (monitoring and repeatability) of each type of lighting system recorded enabled the physical parameters Palaeolithic light to be quantified. These data enrich our knowledge of Palaeolithic activities developed in the darkness of caves through the application of GIS studies (palaeo-speleological behaviours, visibility of art, techniques used, etc.), and lead to a more realistic and attractive dissemination of the underground heritage in digital environments and physical replicas. These experimental activities were carried out in a natural cave, without archaeological remains, located in Lekeitio (Bizkaia, Spain). Thanks to the Before-Art project team (dir. Diego Garate, University of Cantabria), that manages and has conditioned this cave, the cave has the necessary equipment to carry out the experimentation and light monitoring. The main tools used for the monitoring of the fires were a luminous flux temperature meter with built-in luxmeter, sensors to record temperature, CO2 and humidity, thermocouples, a reflectometer and a spectrophotometer. Fire residues were also recorded from the experimental activity and then mapped for comparison with archaeological records using GIS and to obtain reference samples. During these experimental activities, we collaborated closely with the experimental archeologist Jean Leblanc (Université de Toulouse).

5. Analyses, integration of data and synthesis: The R statistical package for statistical computation and graphics was used to explore the data set, as well as a heatmap tool in QGIS® to create a Kernel Density Map through vector point layers with the location of the lighting traces (archaeological and experimental residues), to observe the different distribution patterns and their relationship with other underground elements (structures, rock art, etc.) and with different human movements inside the cave. Bayesian analysis (©Oxcal 4.4.) was used for data management of the dating results. At the end of the project, the results will be interpreted in light of the available literature on palaeolithic underground activities. Lastly, the role of firelight in palaeolithic underground behaviour will be assessed based on the results obtained throughout the different methodological phases, with emphasis on the importance of light in the performance of the first symbolic activities in the caves and on its economic and socio-cultural implications beyond purely utilitarian aspects.

## Results and discussion

In the following, we will present some results obtained in the framework of the postdoctoral project "Archaeology of the Light" during the year 2021–2023.

### Wood for light. Beyond a functional aspect?

**Figure 3.**
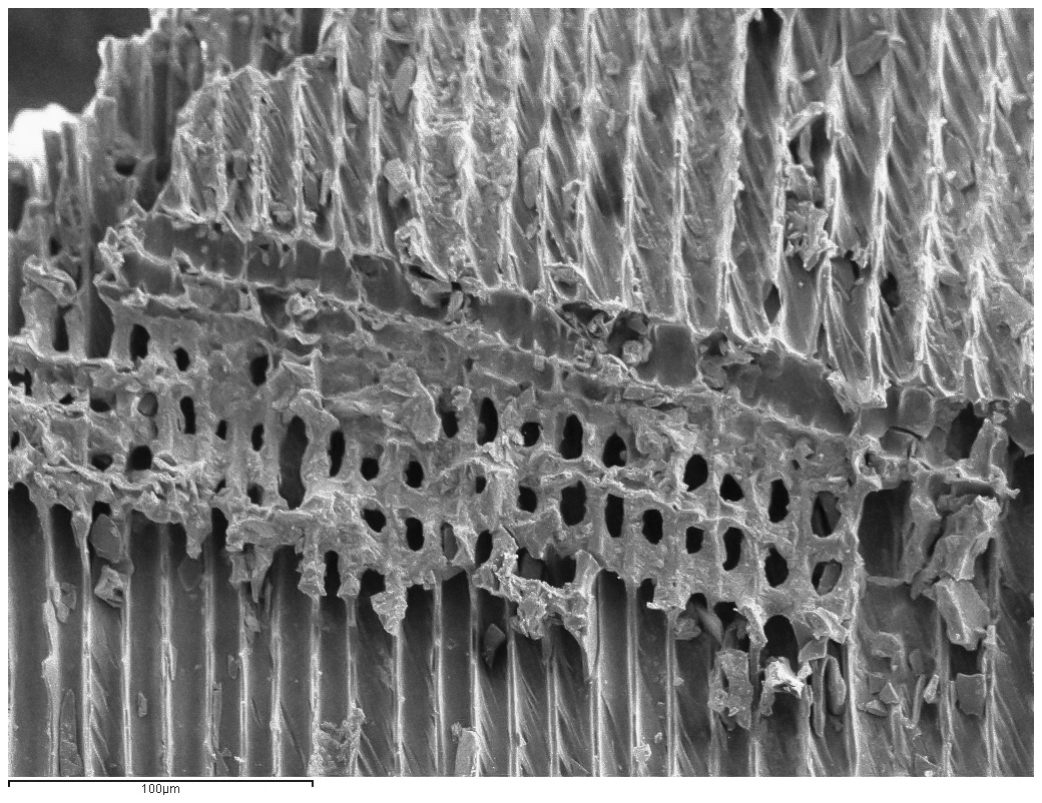
SEM photograph of *Pinus* tp. *sylvestris-nigra* charcoal from a paleolithic lamp.

Inside Nerja cave (Málaga, Spain), we located remains of three different lighting systems (torches, fires and fixed lamps). The anthracological study of 336 charcoals from the woody fuel used in these prehistoric lighting resources shows a preference for the use of *Pinus sylvestris*- *nigra* (51.79%) ( Figure 3). Other taxa were also identified ^2^: *Pinus* cf. tp. *pinea*- *pinaster* (2.38%), *Pinus* sp. (3.87%), conifer (12.80%), Leguminosae (4.16%), Prunus sp. (0.30%), cf. Ulex sp. (0.30%), angiosperm (2.98%), indeterminable charcoals (7.44%) and pith remains (0.30%). In summary, we characterized 4 different types of taxa: *Pinus* tp. *sylvestris*- *nigra* (scots pine and black pine), *Pinus* cf. tp. *pinea*- *pinaster* (maritime pine and stone pine), *Prunus* sp. (blackthorn) and *Ulex* sp. (gorse). Other charcoals were defined with a lower degree of identification; they could correspond to the taxa mentioned above, or to others not identified at the taxonomic level. This was mainly due to the low consistency of most of the charcoals analyzed, which disintegrated when trying to prepare an optimal section for examination, and to the poor state of preservation of some of the remains.

*Pinus* tp. *sylvestris*- *nigra* is the most common anthracological type found in the interior of caves and is linked to artificial lighting in the Upper Palaeolithic, specifically in the pre-Magdalenian period ( Medina-Alcaide *et al*., 2021). In some caves, it has even been documented almost exclusively: in the Chauvet cave, for instance ( Théry-Parisot & Thiébault, 2005; Théry-Parisot *et al*., 2018), only one fragment of *Rhamnus* cf. (buckthorn family) was identified compared to 292 fragments of *Pinus sylvestris*. Environmental availability in the surroundings of the cave, the suitability of this wood for lighting the cave due to its resin content and other cultural aspects have been proposed as the reasons for this preferential choice ( Théry-Parisot & Thiébault, 2005; Théry-Parisot *et al*., 2018).

In Nerja cave all the identified woods used to light fires were also determined in the habitat shelters in the access areas of the cave ( Badal, 1996), as well as fitting in with other palaeoenvironmental studies in the south of the Iberian Peninsula, such as the palaeopollen sequences from Bajondillo cave (Torremolinos, Málaga) ( López-Saez *et al*., 2007), from Gorham cave (Gibraltar) ( Carrión *et al*., 2008), from the Padul peat bog (Granada) ( Carrión, 2012; Pons & Reille, 1988), etc. In other words, the woody species used for illumination were **available in the cave environment** and do not seem to come from more distant areas. Specifically, all the radiocarbon dates we have carried out on Pinus tp. *sylvestris*- *nigra* charcoals have a Palaeolithic chronology (>15000 years ago), and in no case a Holocene chronology. These palaeoenvironmental data are in line with the anthracological study carried out in the stratigraphic sequence of the Vestibule (external room of the cave) ( Badal, 1996), where, although this wood is present throughout the stratigraphic sequence from the Gravettian to the Epipalaeolithic, from the Upper Magdalenian onwards it loses its predominant character and is present in low percentages. Also, thanks to our results, we can suggest that the choice of *Pinus* tp. *sylvestris*- *nigra* wood for lighting-related activities seems to be **a cross-cultural aspect, shared by palaeogroups over a long period of time (30,000 years)**. In particular, the choice of this type of wood for lighting has been recorded from the Aurignacian to the Late Magdalenian in Nerja cave. Likewise, this aspect, reiterative over time, has been indicated for the preferential use of *Pinus sylvestris* in the Chauvet cave, although only for two different periods of occupation, the Aurignacian and the Gravettian ( Théry-Parisot *et al*., 2018).

Our data also agree with the socio-economic deduction proposed in the previous anthracological study of Nerja cave ( Badal, 1996) regarding the avoidance of stone pine wood as fuel, in view of the scarce fragments of this carbonised wood recognised throughout the external stratigraphic sequence and the abundance of pine cone bracts and pine nut shells from this tree. This led Badal and colleagues to consider the exploitation of this tree for food rather than fuel ( Badal, 1996; Badal *et al*., 2014). Our results confirm this management of plant resources by Palaeolithic groups and the prior **planning of wood used** as fuel depending on the activity. Specifically, the fuel used for lighting is mainly *Pinus* tp. *sylvestris*- *nigra*, with *Pinus* tp. *pinea*- *pinaster* being very sparsely represented (2.38%).

The manual collection of the remains could also be one of the causes of the overestimation of some taxa over others ( Chabal, 1997). In fact, when we applied a more exhaustive collection of the carbonised remains (by flotation), for example in the case of the Ledge of Horses in the Atxurra cave ( Garate *et al*., 2023), a greater number of taxa were found. Their variability is not high, however, and the representativeness of some taxa over others continues to be eloquent in favour of one or, at most, two determinations. In this sense, ethnographic studies show that monofunctional fires, i.e. those linked to a specific activity (such as lighting in our case), have a higher degree of fuel selection ( Henry *et al*., 2018).

Concerning the taphonomic alterations of the analysed charcoals, we observed different anomalies linked to the combustion process, such as vitrification (54.14%) and shrinkage cracks (32.41%). Vitrification is related to several factors, some of which may fit with the particularities of the context of origin of our charcoals, for example: a. the sudden interruption of combustion as a trigger for vitrification ( Carrión, 2005) could be connected, in our context, to the gradual detachment of the charcoal from the torches (during the pyrolysis stage); b. the burning of **small branches** ( Marguerie & Hunot, 2007) could correspond to the diameter of wood suitable for transport to deep contexts of the caves, as well as its suitability for the manufacture of torches; c. the burning of dense wood with high **resin content** ( Py-Saragaglia & Ancel, 2006; Scheel-Ybert, 1998) could be linked with the taxonomic identification of most of our charcoal samples (pine and conifer wood); d. the aerobic degradation of the wood prior to combustion ( Henry, 2011) could be linked to the **use of dead wood** derived from natural pruning for lighting ( Théry-Parisot & Thiébault, 2005; Théry-Parisot *et al*., 2018). Concerning the last point, our identification of several samples with a strongly altered structure (44.14%) and with low consistency (hyperfragmentation) could also indicate a preferential use of dead wood, which is easier to collect and ignite, for artificial lighting ( Asouti & Austin, 2005; Scheel-Ybert, 2001; Théry-Parisot, 2001). In addition, this preference would imply a strong **seasonal component for the development of subterranean activity**, outside the snow season, **or planning of the activity** by taking into account the necessary drying time of the wood (at least two years) ( Théry-Parisot *et al*., 2018). Only dried wood guarantees a homogeneous and persistent combustion of flammable gases. However, in our experimental activities, we obtained good results with a slightly shorter dehydration time in a dry and sheltered environment.

**Figure 4.**
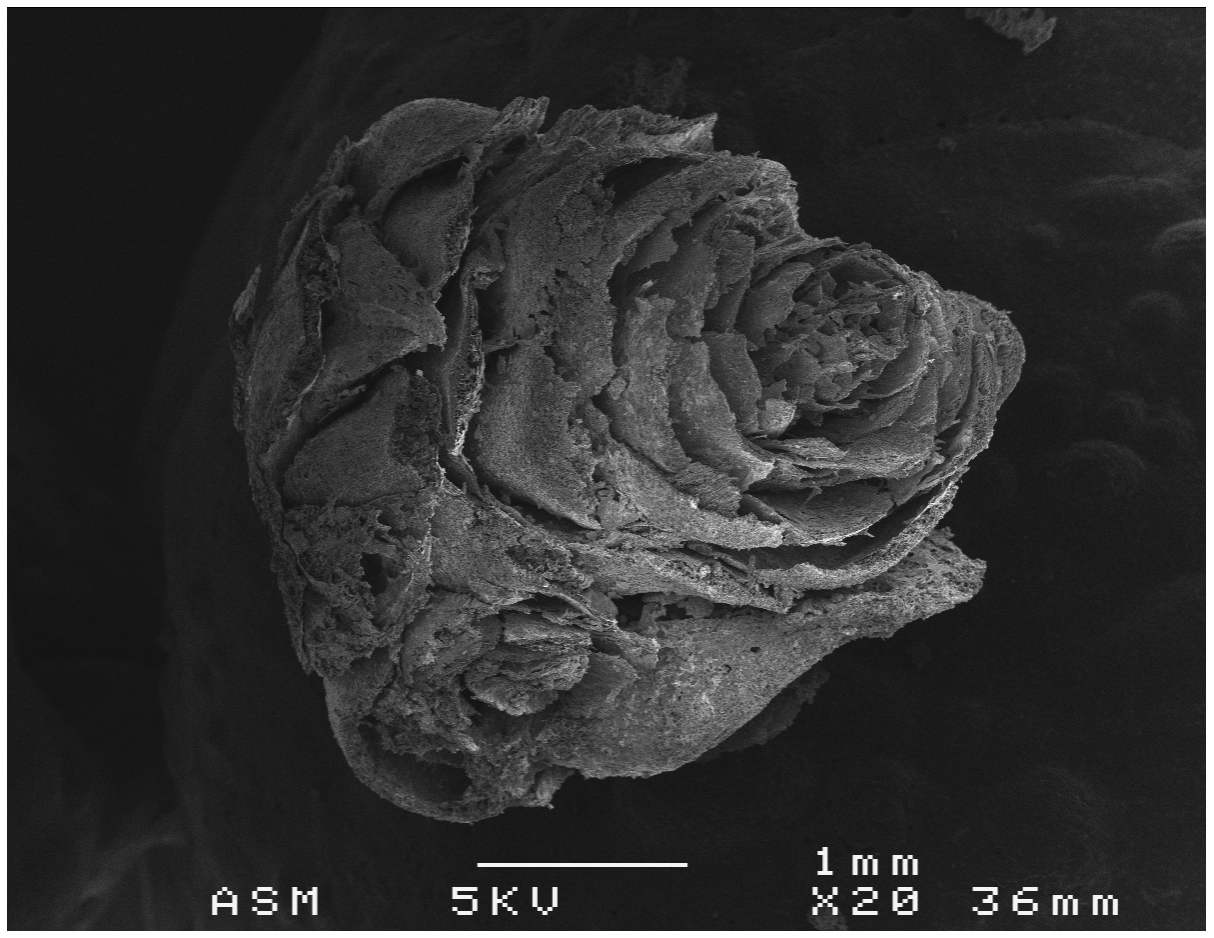
SEM photograph of *Pinus* sylvestris buds.

Also related to this seasonal aspect, we must highlight the vegetative buds of *Pinus sylvestris* located in the internal context of the Nerja cave and related to Gravettian charcoals (27000 years ago) linked to the use of wooden torches ( Figure 4) ( Medina *et al*., 2015). This element is unique in similar contexts. These slightly resinous buds develop at the ends of branches during autumn and winter ( Ruiz de la Torre, 2006). If the wood had been harvested when green, we could infer some seasonal data about the time of year when the visit may have taken place; however, the data obtained to date suggest the harvesting of dead or dry wood, so these shoots may have been part of the fuel collected after natural pruning. However, the discovery of these remains indicates that **the fixed fires or torches could include other plant elements** apart from woody fuel, such as these resin-rich shoots.

Finally, the charcoals examined show brownish stains (22.07%). Based on experimental observations, we attribute these stains to the partial and incomplete burning of the wood. This likely occurred when charcoal fell from the torches before complete combustion; some remains could also be due to partial combustion of a fixed fire.

In addition, traces of reaction wood ( Figure 3) were identified (31.72%), together with some knots (1.38%). These alterations suggest that the firewood used must have come from a set of **thin** **branches**. The spatial context of the samples (internal context of caves, often of complex transit) and the suitability of this type of wood for the manufacture of torches, conditioned the use of thin wood. In addition, previous studies proposed this choice based on the size of the preserved wood remains in some caves, for example, woody imprints found in the clay from the floor of the Chauvet cave ( Clottes, 2001), or wood branches preserved under the concretion in the Aldène cave ( Galant *et al*., 2007).

At this point, we should point out that we tried to carry out a dendroanthracological analysis of the charcoals, although the absence of bark in all the samples (presence of the last ring), the small size and low consistency of the samples severely limited the results in this respect. Of the remains analysed in Nerja cave, only 9 fragments were found to have moderate ring curvature, 21 had strongly curved log rings and the vast majority (259) were classified as having indeterminate ring curvature. This was determined by observing the degree of curvature of the rings in the cross-section of the charcoal ( Marguerie & Hunot, 2007). The high frequency of charcoals with indeterminate curvature considerably limits the representativeness of the results obtained. However, most of the determinable curvatures we observed present pronounced curvatures linked to a position of the charcoal fragment close to the pith or related to **small diameter twigs**; to a lesser extent they are consistent with moderate curvatures related to larger branches in relation to the previous ones or coals further away from the central axis; on no occasion did we observe weak ring curvatures linked to the combustion of large trunks.

The presence of hyphae was also recorded (32.76%), preferably post-combustion (white). This anomaly is linked to the endokarst microbial activity and to the high humidity. As far as possible, we preferred not to send any charcoal with this type of alteration to be dated, as it has been related to the possible rejuvenation of some radiocarbon dates obtained in similar contexts (for example, in the Tito Bustillo cave - Fortea, 2007).

In summary, beyond the species, the state or the size of the wood chosen as fuel, various ethnobotanical studies also show that historical and cultural conditioning factors influenced the selection of wood for firewood, even when other species were abundant in the vegetation ( Henry *et al*., 2009; Henry *et al*., 2018; Lavrillier, 2007; Zapata & Peña-Chocarro, 2003). At the same time, socio-economic factors have been pointed out in the choice of fuel, such as the previously cited example of Nerja Cave ( Badal, 1996; Badal *et al*., 2014). While the cultural and symbolic conditioning factors are difficult to ascertain from the archaeological record, they must nevertheless be taken into account, particularly in the archaeological context examined, which is often far removed from basic subsistence activities and subject to behaviours of a symbolic or ritual nature.

### Prehistoric cave life through soot and charcoal trails

Through the C14-AMS dating of 53 of these charcoals, we constructed a high-precision Bayesian model to identify the **minimum number of prehistoric visits** to the interior of Nerja cave ( Medina-Alcaide *et al*., 2023). The different phases were defined according to the presence of archaeological materials (and not only through the radiocarbon dating results) of each of the proposed phases in the stratigraphic deposits of the entrance rooms of the cave, or, failing that, to the presence of this type of cultural material in the regional context. For this purpose, using OxCal4.4, we sequenced these suggested Phases, limited by Boundaries determining the temporal distributions associated with the changes of phase. We also used the Outlier Charcoal model ( Bronk Ramsey, 2009) to properly down-weight outlier samples, taking into account the long life of the charcoal samples. This allowed us first to confirm the statistical plausibility of the model, then to determine the start date (beginning of the boundary) and the end date (end of the boundary) for the different phases of human presence within the cave, their duration and the transition period between phases ( Bronk Ramsey, 1995; Bronk Ramsey, 2001; Bronk Ramsey & Lee, 2013; Morrell, 2019).

**Figure 5.**
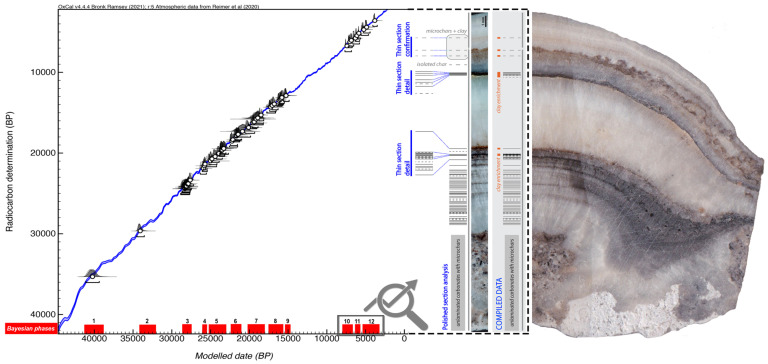
Phases of visits inside of Nerja cave identified by Bayesian model and micro-layers of soot.

The Bayesian model suggests at least **12 distinct phases** of visits to the interior of Nerja cave between **41,218 and 3299 cal BP**, with an agreement index (Aoverall) of 98. These phases of visits to the interior of the cave correspond to the specific chronocultural periods for the prehistoric regional context and with transitional periods: Early Aurignacian (phase 1), Recent Aurignacian (phase 2), Gravettian (phase 3), Lower Solutrean (phase 4), Middle Solutrean (phase 5), Upper Solutrean (phase 6), transition between the Upper Solutrean and Lower Magdalenian, and Lower Magdalenian (phase 7), Middle Magdalenian (phase 8), Upper Magdalenian (phase 9), Early Neolithic (phase 10), Recent Neolithic (phase 11) and Copper Age (phase 12) ( Figure 5).

There are 12 possible transition intervals between the different phases. One of them, separating phases 9–10 (between the Upper Magdalenian and the Early Neolithic), has a notable chronological amplitude (> 6000 years). There are however 8 transition periods that could correspond to 0 years ( **almost-continuous phases**, taking into account the minimum values), in particular, those between phases 1–2 (both could be included in the Aurignacian, the former probably belonging to the ancient phase and the latter to the evolved stage), 4–5 (between the Lower Solutrean and the Middle Solutrean), 5–6 (between the Solutrean and the Middle Solutrean), 6–7 (around the Upper Solutrean and the transition between the Upper Solutrean and Lower Magdalenian), 7–8 (between the Lower Magdalenian and Middle Magdalenian), 8–9 (between the Middle Magdalenian and Upper Magdalenian), 10–11 (between the Early Neolithic and Recent Neolithic), and 11–12 (between the Recent Neolithic and Chalcolithic).

Through the identification, microcounting and dating of micro-layers of soot (fulinochronological analysis - Vandevelde *et al*., 2017; Vandevelde *et al*., 2018; Vandevelde *et al*., 2020; Vandevelde, 2021-) present inside a small stalagmite, we specified the minimum number of visits for the last three phases identified in the Bayesian analysis. This multianalytic approach was developed in collaboration with S. Vandevelde, E. Pons-Branchu and H. Valladas (Laboratoire des Sciences du Climat et de L’Environnement, LSCE/IPSL, CEA-CNRS-UVSQ, Université Paris-Saclay). The chronology of the soot microlayers found in the stalagmite was determined by the C14-AMS dating of CaCO3 layers deposited before and after them, following the methodology previously used in other studies on Nerja cave ( Pons-Branchu *et al*., 2022; Sanchidrián *et al*., 2017; Valladas *et al*., 2017). The soot deposits at the base of the stalagmite were deposited between 7562 and 6736 cal. BP; the soot microlayers from the upper level of the stalagmite were deposited between 6836 and 2998 cal. years BP assuming 0% of DCP for the calibration of the dating.

A minimum of **64 occupations were identified between 7000 and 3000 years** (a Minimum Number of Occupations—MNO that can be increased to 82 if we also count uncertain soot films and microcharcoal alignments; this second case will be included in brackets from now on). If we relate these data to the phases determined by Bayesian analysis, we can state that at least 58 (71) different occupations occurred in phases 10 and 11 (Early Neolithic and Recent Neolithic) with no apparent hiatus between these two phases (a result that is consistent with the Bayesian model from charcoal), and at least 6 (8) visits in phase 12 (Copper Age). The Figure 5 (modified from Medina-Alcaide, Medina-Alcaide *et al*., 2023) shows different phases of visits to the interior of Nerja cave identified by a Bayesian model from charcoal (in red) and image of the different micro-layers of soot of the stalagmite, which increases the minimum number of occupations to at least 64 for the last 3 Bayesian phases. The succession of soot films in the carbonates is represented as barcode diagrams. Bars represent soot films and dashed lines represent probable soot films. The long vertical grey line next to the barcode represents speleothem total thickness.

**Figure 6.**
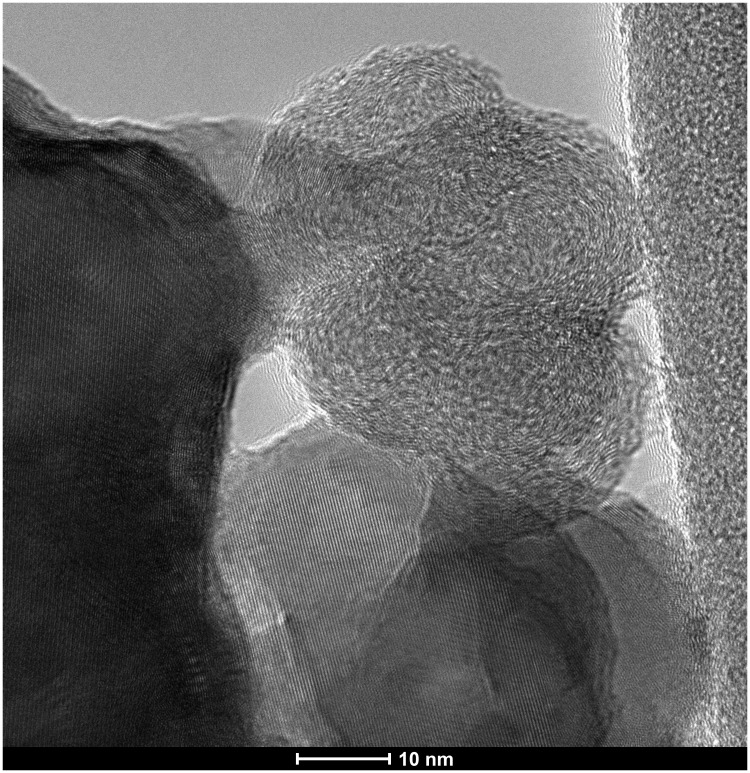
TEM-EDX photograph of nanometric spherical particles of prehistoric fossilized soot.

The identification of the soot remains inside the stalagmite as microlayers was characterised by TEM-EDX (following Pawlyta & Hercman, 2016) and Raman analysis (following Sadezky *et al*., 2005 and Deldicque *et al*., 2016). TEM–EDX observations revealed spherical carbon particles of soot aggregates ( Figure 6). In Nerja Cave, similar particles were observed inside a Palaeolithic fixed lamp in the upper galleries ( Medina-Alcaide *et al*., 2019), but soot residues were also located in another speleothem fragment inside the cave ( Del Rosal, 2016; Pons-Branchu *et al.*, 2022). Microscopic observation also revealed clay deposits together with microcharcoal and soot levels, suggesting that clay was not brought in by percolation at different times of the stalagmite formation but that the human visits to the cave contributed to the suspension of clays that re-deposited in the stalagmite together with the particulates from wood combustion (soot and microcharcoal).

### Reliving paleolithic light for a better understanding of paleolithic cave art

**Figure 7.**
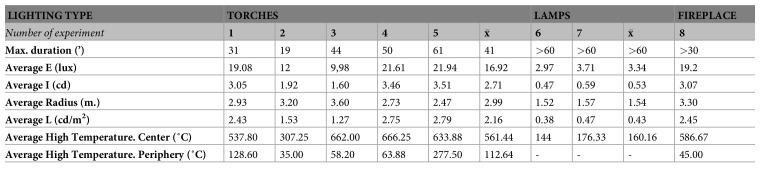
Average values of the parameters measured in each experiment with paleolithic torches, lamps and fireplaces.

**Figure 8.**
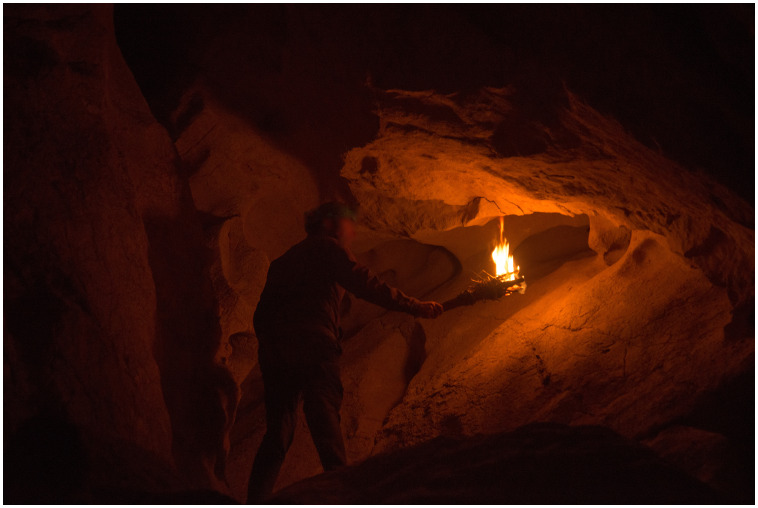
Photograph of the experimental recreation of paleolithic lighting with torches.

The **experimental replication of Palaeolithic light** was monitored in an endokarst environment in order to resemble the humidity, temperature and moisture conditions of the Palaeolithic context as closely as possible. The physical parameters obtained for the three Palaeolithic lighting systems are given in Figure 7– Figure 8. These light resources were constructed on the basis of an exhaustive compilation of the existing literature on the subject, which also included archaeological data obtained as part of our doctoral thesis and postdoctoral study.

The functioning of five wood torches was evaluated in a cave context (experiments 1–5 ( Figure 7). They were all made from branches of dry juniper wood 1.2 cm thick that were joined together. This type of structure was chosen because it fits with the archaeological data and the form of the few prehistoric torches that have been preserved. For example, it matches the thickness of the remains of branches that have been found in the caves of Aldène and Réseau Clastres and with the morphology of a Hallstatt torch ( Medina-Alcaide *et al*., 2021). Birch bark was included among the juniper branches as a kind of tinder to start combustion. Similarly, the wood was broken up to facilitate combustion (through easier oxygenation) and impregnation with non-ligneous fuel. Thus, pine resin, animal fat, or a combination were added in some cases to assess the function of the torches with those fuel types.

For fireplaces, the wood fuel was thin branches of juniper and oak wood in a dry state, arranged in a tepee-shaped structure. Birch bark was used to start the fire. This was placed inside the combustion structure. The fireplace was 23 cm in diameter and 7 cm high (measured before lighting), had no boundary structure, and was lit on a clay substrate. This experiment was carried out at a distance of 80 cm from the closest wall and 1.60 m from the ceiling in order to better assess the reflection of the light.

The lamps used in the experiments were replicas of the lamp from La Mouthe Cave (Dordogne, France) ( Berthelot, 1901). This lamp was made of sandstone, 17 cm long and 12 cm wide, and had a concavity with ≈150 cm ^3^ capacity. Bovid marrow was used as the main fuel, with three woody wicks composed of dried and crushed juniper wood. They were arranged in a tepee shape in the center of the active part of the piece. Pine resin was added in one experiment (number 7) to assess its lighting benefits.

**Figure 9.**
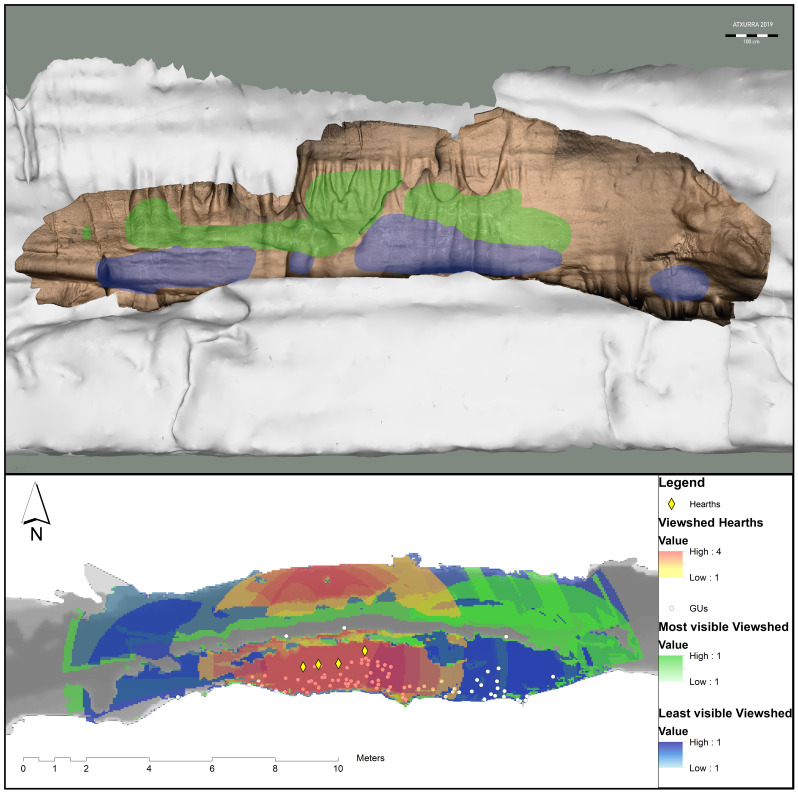
Viewshed analysis of Sector J of Atxurra cave.

**Thanks to the physical parameters of Palaeolithic light thus deduced, we developed pioneer methodologies in GIS environments for the quantitative determination of the degree of visibility, gauging and difficulty of access to Palaeolithic Art** ( Intxaurbe *et al*., 2021; Intxaurbe *et al*., 2022). The Figure 9 show the 3D restitution of the rock art combined with the 3D model of the cave, showing that the panel is located over a cornice, and (B) the viewsheds of the Graphic Unites in Sector J using ArcMap in ArcGIS. The most visible figures are in a high panel. The influence of the archaeological context (in these cases the presence of hearths) is also important to make inferences ( Intxaurbe *et al*., 2020: Figure 6). Besides, we took advantage of the potential of GIS (ArcGIS) to design a cutting-edge methodology to quantify the visibility and capacity in sectors with Palaeolithic Art inside caves. This allowed us to estimate the maximum potential audience for Palaeolithic Art, and the optimal location for visibility, by creating a PYTHON script that was integrated into the software, and provided a comparison with quantitative data between different caves. This innovative methodology was successfully applied in three renowned caves in the Basque Country: Santimamiñe, Altxerri and Atxurra. It showed that, in some cases, there may have been prior planning to improve the visibility of some figures. In all cases, the groups of figures are located in deep and hidden parts of the caves, normally in sectors with a limited capacity to hold people, which would be consistent with a restricted communication system ( Intxaurbe *et al*., 2021). 

For example, in the paradigmatic case of the shelf in sector J of Atxurra **, the location of the hearths** at the foot of the rock art ensemble (dated to the same period as the artworks themselves on the adjacent wall) **seems to have been determined to provide visibility**, as the halo of firelight would have illuminated almost the entire wall with paleolithic art in this zone ( Garate *et al*., 2020a; Garate *et al*., 2020b; Medina-Alcaide *et al*., 2021), thus increasing the visibility indices of the figures on the right side of the wall. This is even more prominent for the two main horses of the composition as they feature a number of techniques such as engraving and scraping, and maybe painting.

Secondly, the physical parameters of Palaeolithic light obtained through our archeological and experimental studies were used to propose a scoring methodology to quantify the accessibility of Palaeolithic Art inside the caves. The proposed methodology provides information on the difficulty of access, the estimated time of arrival, the length of the optimal route, etc. ( Intxaurbe *et al*., 2022). We developed a PYTHON script that integrates light and velocity data (experimental tests with professional cavers) for the spatial analysis of the 3D models of the caves from GIS. This spatial study of the paleolithic art in the caves makes it possible to compare the accessibility of the sites with quantitative (and not only qualitative) data, which is essential to investigate which graphical elements are more inaccessible or hidden and why.

This methodology was applied in Atxurra Cave to evaluate the accessibility of all the rock art zone. As a preliminary conclusion, the results obtained seem to show that the artists' choice of panel was directly affected by the difficulty of transit. The Upper Magdalenian groups at Atxurra chose panels that were difficult to access, despite the fact that the options offered by the cave are much wider and less risky. However, the reason for this is unknown: a desire to "hide" this art? evidence of rites of passage? ( Arias, 2009; Owens & Hayden, 1997). In some cases, it seems that the locations in high areas could have been related to more functional reasons, such as giving **visual prominence to certain figures**, as in Sector J. This, as mentioned above, is accentuated by the location of the fires lit in this area of the cave.

Logically, the estimated time to access each sector increases for areas that include constant vertical zones (passable by climbing or traversing). The experience, physical strength or knowledge of the topography of the cave would help to optimise the time needed to access the deep areas.

## Conclusion

The first results obtained within the project entitled the A-Light (funded by the Fyssen Foundation between 2021, and MSCActions PF 2022–2024) confirm the potential of the interdisciplinary study of combustion and lighting remains in caves for the knowledge of prehistoric subterranean activities, including rock art. The exhaustive anthracological analysis of charcoals from fires, including taxonomic, taphonomic and dendroanthracological approaches, suggests that there was a selection of fuel for cave lighting probably linked to its environmental availability, its lighting capabilities and for other economic and cultural reasons. Thanks to a pioneering multiproxy and interdisciplinary approach, the C14-AMS dating, the Bayesian analysis, and the interdisciplinary study of the soot deposits, we have been able to define the useful life of the cave (in the case of Nerja cave we have extended the origin of human occupation of this famous cave by 10000 years) and the minimum number of prehistoric visits to the cave interior. Experimental replication and monitoring of the physical parameters of Palaeolithic light has allowed us to better understand the functioning of these lighting systems, as well as to develop innovative methodologies for the evaluation of the visibility and accessibility of Palaeolithic art inside the caves. In short, these data demonstrate that the *Archaeology of the Light* is here to stay and that it is an essential approach for a holistic understanding of Palaeolithic caves.

## Ethics and consent

Ethical approval and written informed consent were not required.

## Data Availability

No data are associated with this article No data are associated with this article No data are associated with this article

## References

[ref-1] Allain J : Les lampes magdaléniennes de Saint-Martin (Indre). In: *Congrès Préhistorique de France*.1965;178–183.

[ref-2] Arias P : Rites in the dark? An evaluation of the current evidence for ritual areas at Magdalenian cave sites. *World Archaeol.* 2009;41(2):262–294. 10.1080/00438240902843964

[ref-3] Arias P Ontañón R : La Garma: un sitio excepcional, una metodología diferente. In: *Actualidad de la investigación arqueológica en España I (2018-2019): conferencias impartidas en el Museo Arqueológico Nacional.*Subdirección General de Atención al Ciudadano, Documentación y Publicaciones, Madrid:2020;45–64.

[ref-4] Asouti E Austin P : Reconstructing woodland vegetation and its exploitation by past societies, based on the analysis and interpretation of archaeological wood charcoal macro-remains. *Environmental Archaeology.* 2005;10(1):1–18. 10.1179/env.2005.10.1.1

[ref-5] Badal E : La vegetation du Paleolithique superieur et de l’Épipaleolithique aux alentours de la Cueva de Nerja. *Actes du colloque de Périgueux 1995. Supplément à la Rev. d’Archéom.* 1996;171–176.

[ref-6] Badal E Vidal P Sanchidrián J : Pinions to eat? Again? *XVII World UISPP Congress.*Burgos.2014;256.

[ref-7] Beaune SA : Lampes et godets au Paléolithique.Supplément à Gallia Préhistoire 23. Editions du Centre National de la Recherche Scientifique, Paris,1987. Reference Source

[ref-8] Berthelot M : Sur une lampe préhistorique trouvé dans la grotte de La Mouthe. *Comptes Rendus Acad Sci.* 1901;666.

[ref-9] Billson JM Mancini K : Inuit women: their powerful spirit in a century of change.Ed. Rowman Littlefield Publishers, New York,2007. Reference Source

[ref-12] Bronk Ramsey C : Radiocarbon calibration and analysis of stratigraphy: the OxCal program. *Radiocarbon.* 1995;37(2):425–430. 10.1017/S0033822200030903

[ref-11] Bronk Ramsey C : Development of the radiocarbon calibration program. *Radiocarbon.* 2001;43(2A):355–363. 10.1017/S0033822200038212

[ref-10] Bronk Ramsey C : Dealing with outliers and offsets in radiocarbon dating. *Radiocarbon.* 2009;51(3):1023–1045. 10.1017/S0033822200034093

[ref-13] Bronk Ramsey C Lee S : Recent and planned developments of the program OxCal. *Radiocarbon.* 2013;55(2):720–730. 10.1017/S0033822200057878

[ref-14] Carrasco G Fortes-Román FJ Liñán-Baena C : Estudio de las alteraciones del soporte rocoso y de los espeleotemas de la Cueva de Nerja (Málaga) mediante tecnología LIBS. In: Andreo B, Durán JJ: (eds.): *El karst y el hombre: las cuevas como Patrimonio Mundial.*Asociación de Cuevas Turísticas Españolas. Madrid:2016;347–360.

[ref-15] Carrión Y : La vegetación mediterránea y atlántica de la península ibérica: nuevas secuencias antracológicas.Diputación provincial de Valencia, Valencia.2005.

[ref-17] Carrión JS : Paleoflora y paleovegetación de la Península Ibérica e Islas Baleares: Plioceno-Cuaternario.Ministerio Economía y Competitividad, Universidad de Murcia, Murcia,2012. Reference Source

[ref-16] Carrión JS Finlayson C Férnández S : A coastal reservoir of biodiversity for Upper Pleistocene human populations: Palaeoecological investigations in Gorham’s Cave (Gibraltar) in the context of the Iberian Peninsula. *Quat Sci Rev.* 2008;27(23–24):2118–2135. 10.1016/j.quascirev.2008.08.016

[ref-18] Chabal L : Forêts et sociétés en Languedoc (Néolithique final, Antiquité tardive): l'anthracologie, méthode et paléoécologie.Maison des Sciences de l'Homme, Paris,1997.

[ref-20] Clottes J : Contexte archéologique interne. In: G.R.A.P.P: (eds.): *L’art pariétal Paléolithique. Techniques et méthodes d’étude*. CTHS, Paris,1993;49–58.

[ref-21] Clottes J : *La grotte Chauvet: l'art des origines.*Seuil, Paris,2001.

[ref-19] Clottes J Simonnet R : Retour au Réseau Clastres (Niaux Ariege). *Préhistoire Ariégeoise.* 1990;45:51–139.

[ref-23] Deldicque D Rouzaud JN Vandevelde S : Effects of oxidative weathering on Raman spectra of charcoal and bone chars: consequences in archaeology and paleothermometry. *C R Geosci.* 2023;355(G1):1–22. 10.5802/crgeos.186

[ref-22] Deldicque D Rouzaud JN Velde B : A Raman–HRTEM study of the carbonization of wood: a new Raman-based paleothermometer dedicated to archaeometry. *Carbon.* 2016;102:319–329. 10.1016/j.carbon.2016.02.042

[ref-24] Delluc B Delluc G : L´éclairage. In: Leroi-Gourhan A, Allain J. (eds.): *Lascaux inconnu.*Centre National de la Recherche Scientifique, Paris:1979;121–142.

[ref-25] Del Rosal Y : Análisis, impacto y evolución de biofilms fotosintéticos en espeleotemas. El caso de la Cueva de Nerja. PhD tesis. Universidad de Málaga,2016. Reference Source

[ref-26] Donais MK Vandenabeele P : Portable spectroscopy for on-site and *in situ* archaeology studies. *Portable Spectroscopy and Spectrometry.* 2021;1:523–544. 10.1002/9781119636489.ch44

[ref-27] Fortea J : 39 edades 14C AMS para el arte paleolítico rupestre en Asturias. *Excavaciones arqueológicas en Asturias, 1999-2002.* 2007;91–102.

[ref-28] Galant P Ambert P Colomer A : Les vestiges d’éclairages préhistoriques de la galerie des Pas de la grotte d’Aldène (Cesseras Hérault). *Bulletin du Musée d'anthropologie préhistorique de Monaco.* 2007;47:37–80.

[ref-29] Garate D : Más allá de la imagen: el arte parietal paleolítico en su contexto arqueológico. *Kobie Serie Anejo.* 2017;16:149–162.

[ref-30] Garate D Rivero O Rios-Garaizar J : The cave of Atxurra: a new major Magdalenian rock art sanctuary in Northern Spain. *J Archaeol Sci Rep.* 2020a;29: 102120. 10.1016/j.jasrep.2019.102120

[ref-31] Garate D Rivero O Rios-Garaizar J : Modelled clay animals in Aitzbitarte iv cave: a unique Palaeolithic rock art site in the Cantabrian Region. *J Archaeol Sci Report.* 2020b;31: 102270. 10.1016/j.jasrep.2020.102270

[ref-32] Garate D Rivero O Rios-garaizar J : Unravelling the skills and motivations of magdalenian artists in the depths of Atxurra Cave (Northern Spain). *Sci Rep.* 2023;13(1): 17340. 10.1038/s41598-023-44520-w 37833336 PMC10575969

[ref-33] Henry A : Paléoenvironnements et gestion des combustibles au Mésolithique dans le sud de la France: anthracologie, ethnoarchéologie et exp ´erimentation. Tesis doctoral, Université Nice Sophia Antipolis, Nice,2011. Reference Source

[ref-34] Henry A Théry-Parisot I Voronkova E : La gestion du bois de feu en forêt boréale: problématique archéo-anthracologique et étude d'un cas ethnographique (Région de l'Amour, Sibérie).In: I. Théry-Parisot, S. Costamagno & A. Henry (eds.): *Gestion des combustibles au paléolithique et au mésolithique Nouveaux outils, nouvelles interpretations/Fuel Management during the Palaeolithic and Mesolithic Periods New tools, new interpretations.*BAR S1914, Oxford,2009;17–37. Reference Source

[ref-35] Henry A Zavadskaya E Alix C : Ethnoarchaeology of fuel use in northern forests: towards a better characterization of prehistoric fire-related activities. *Ethnoarchaeology.* 2018;10(2):99–120. 10.1080/19442890.2018.1510601

[ref-36] Hernanz A : Raman spectroscopy of prehistoric pictorial materials.In: P. Bueno-Ramírez & P.G. Bahn (eds.): *Prehistoric Art as Prehistoric Culture. Studies in Honour of Professor Rodrigo de Balbín-Behrmann.*Archeopress, Oxford,2015;11–20. Reference Source

[ref-37] Intxaurbe I Arriolabengoa M Medina-Alcaide MÁ : Quantifying accessibility to Palaeolithic rock art: methodological proposal for the study of human transit in Atxurra Cave (Northern Spain). *J Archaeol Sci.* 2021;125: 105271. 10.1016/j.jas.2020.105271

[ref-38] Intxaurbe I Garate D Arriolabengoa M : Application of line of sight and potential audience analysis to unravel the spatial organization of palaeolithic cave art. *J Archaeol Method Th.* 2022;29(4):1158–1189. 10.1007/s10816-022-09552-y

[ref-39] Intxaurbe I Rivero O Medina-Alcaide MÁ : Hidden images in Atxurra Cave (Northern Spain): a new proposal for visibility analyses of palaeolithic rock art in subterranean environments. *Quatern Int.* 2020;566–567:163–170. 10.1016/j.quaint.2020.04.027

[ref-41] Jaubert J Genty D Valladas H : The chronology of human and animal presence in the decorated and sepulchral cave of Cussac (France). *Quatern Int.* 2016b;432:5–24. 10.1016/j.quaint.2016.01.052

[ref-40] Jaubert J Verheyden S Genty D : Early Neanderthal constructions deep in Bruniquel Cave in southwestern France. *Nature.* 2016a;534(7605):111–114. 10.1038/nature18291 27251286

[ref-42] Lavrillier A : Gestion duelle de l'espace à long terme chez les évenks éleveurs de rennes et chasseurs des Monts Stanovoï: interférences ou cohérences des zones sauvages et humanisées.In: S. Beyries y V. Vaté (eds.): *Les civilisations du renne d'hier et aujourd'hui. Approches ethnohistoriques, archéologiques et anthropologiques.*APDCA, Antibes,2007;65–88.

[ref-43] López-Sáez JA López-García P Cortés M : Paleovegetación del Cuaternario reciente: Estudio arqueopalinológico.In: M. Cortés (ed.): Cueva Bajondillo (Torremolinos). *Secuencia cronocultural y paleoambiental del Cuaternario reciente en la Bahıa de Málaga*. Diputación de Málaga, Málaga,2007;139–156.

[ref-44] Marguerie D Hunot JY : Charcoal analysis and dendrology: data from archaeological sites in north-western France. *J Archaeol Sci.* 2007;34(9):1417–1433. 10.1016/j.jas.2006.10.032

[ref-45] Medina-Alcaide MÁ : Iluminando la oscuridad de las cuevas con manifestaciones gráficas paleolíticas: una visión integral e interdisciplinar del contexto arqueológico interno y de los carbones de madera. Doctoral thesis. Universidad del País Vasco (UPV/EHU). Vitoria-Gasteiz,2019. Reference Source

[ref-46] Medina-Alcaide MÁ Sanchidrián JL Zapata L : Lighting the dark: Wood charcoal analysis from Cueva de Nerja (Málaga, Spain) as a tool to explore the context of Palaeolithic rock art. *C R Palevol.* 2015;14(5):411–422. 10.1016/j.crpv.2015.03.010

[ref-47] Medina-Alcaide MÁ Cabalín LM Laserna J : Multianalytical and multiproxy approach to the characterization of a paleolithic lamp. An example from Nerja cave (South of Iberian Peninsula). *J Archaeol Sci Report.* 2019;28: 102021. 10.1016/j.jasrep.2019.102021

[ref-49] Medina-Alcaide MÁ Garate D Intxaurbe I : The conquest of the dark spaces: an experimental approach to lighting systems in paleolithic caves. *PLoS One.* 2021;16(6): e0250497. 10.1371/journal.pone.0250497 34133423 PMC8208548

[ref-48] Medina-Alcaide MÁ Garate D Ruiz-Redondo A : Beyond art: the internal archaeological context in Paleolithic decorated caves. *J Anthropol Archaeol.* 2018;49:114–128. 10.1016/j.jaa.2017.12.005

[ref-50] Medina-Alcaide MÁ Vandevelde S Quiles A : 35,000 years of recurrent visits inside Nerja cave (Andalusia, Spain) based on charcoals and soot micro-layers analyses. *Sci Rep.* 2023;13(1):5901. 10.1038/s41598-023-32544-1 37041224 PMC10090096

[ref-51] Morrell B : La cronología como medio de interpretación social: los contextos funerarios del NE de la península ibérica entre finales del V inicios del IV milenio cal BC. Doctoral dissertation, Universitat Autònoma de Barcelona,2019. Reference Source

[ref-52] Owens DA Hayden B : Prehistoric rites of passage: a comparative study of transegalitarian hunter-gatherers. *J Anthropol Archaeol.* 1997;16(2):121–161. 10.1006/jaar.1997.0307

[ref-53] Papadopoulos C Moyes H : The Oxford Handbook of Light in Archaeology. Oxford Handbooks,2021. 10.1093/oxfordhb/9780198788218.001.0001

[ref-54] Pastoors A Lenssen-Erz T Ontañón R : With the back to the art. Context of Pleistocene cave art. *Quatern Int.* 2017;430:1–4. Reference Source

[ref-55] Pawlyta M Hercman H : Transmission Electron Microscopy (TEM) as a tool for identification of combustion products: application to black layers in speleothems. *Ann Soc Geol Pol.* 2016;86(2):237–248. 10.14241/asgp.2016.004

[ref-56] Pons A Reille M : The Holocene and Upper Pleistocene pollen record from Padul (Granada, Spain): a new study. *Palaeogeogr Palaeoclimatol Palaeoecol.* 1988;66(3–4):243–263. 10.1016/0031-0182(88)90202-7

[ref-57] Pons-Branchu E Barbarand J Caffy I : U-series and radiocarbon cross dating of speleothems from Nerja Cave (Spain): evidence of open system behavior. Implication for the Spanish rock art chronology. *Quaternary Sci Rev.* 2022;290: 107634. 10.1016/j.quascirev.2022.107634

[ref-58] Py-Saragaglia V Ancel B : Archaeological experiments in fire-setting: protocol, fuel and anthracological approach. *BAR International Series S.* 2006;1483:71–82. Reference Source

[ref-59] Ruiz de la Torre J : Flora Mayor. Organismo Autónomo de Parques Nacionales, Madrid,2006. Reference Source

[ref-63] Sadezky A Muckenhuber H Grothe H : Raman microspectroscopy of soot and related carbonaceous materials: spectral analysis and structural information. *Carbon.* 2005;43(8):1731–1742. 10.1016/j.carbon.2005.02.018

[ref-60] Sanchidrián JL Valladas H Medina-Alcaide MÁ : New perspectives for ^14^C dating of parietal markings using CaCO _3_ thin layers: an example in Nerja cave (Spain). *J Archaeol Sci Rep.* 2017;12:74–80. 10.1016/j.jasrep.2017.01.028

[ref-61] Scheel-Ybert R : Stabilité de l’écosystème sur le litoral sud-est du Brésil à l’Holocène Supérieur (5500-1400 ans BP). Tesis doctoral, Université Montpellier II Sciences et Techniques du Languedoc, Montpellier,1998.

[ref-62] Scheel-Ybert R : Man and vegetation in southeastern Brazil during the late Holocene. *J Archaeol Sci.* 2001;28(5):471–480. 10.1006/jasc.2000.0577

[ref-66] Théry-Parisot I : Economie des combustibles au paléolithique: expérimentation, taphonomie, anthracologie. CNRS, Paris,2001. Reference Source

[ref-64] Théry-Parisot I Thiébault S Delannoy JJ : Illuminating the cave, drawing in black: wood charcoal analysis at Chauvet-Pont d'Arc. *Antiquity.* 2018;92(362):320–333. 10.15184/aqy.2017.222

[ref-65] Théry-Parisot I Thiébault S : Le pin (Pinus sylvestris): préférence d'un taxon ou contrainte de l'environnement? étude des charbons de bois de la grotte Chauvet. *Bull Soc Prehist Fr.* 2005;102(1):69–75. 10.3406/bspf.2005.13338

[ref-67] Trimmis KP : Paperless mapping and cave archaeology: a review on the application of DistoX survey method in archaeological cave sites. *J Archaeol Sci Rep.* 2018;18:399–407. 10.1016/j.jasrep.2018.01.022

[ref-68] Valladas H Pons-Branchu E Dumoulin JP : U/Th and ^14^C crossdating of Parietal Calcite deposits: application to Nerja Cave (Andalusia, Spain) and future perspectives. *Radiocarbon.* 2017;59(6):1955–1967. 10.1017/RDC.2017.120

[ref-69] Vandevelde S : Les rythmicités d’occupation d’un site au sein d’un territoire. Approche diachronique.In: L. Slimak, Y. Giraud, L. Metz & P. Yvorra (eds.): *Mandrin. Des Derniers Néandertaliens Aux Premiers Hommes Modernes en France Méditerranéenne. A&t 4*. MMSH,2021;694–709.

[ref-70] Vandevelde S Brochier JÉ Desachy B : Sooted concretions: a new micro-chronological tool for high temporal resolution archaeology. *Quatern Int.* 2018;474:103–118. 10.1016/j.quaint.2017.10.031

[ref-71] Vandevelde S Brochier JÉ Petit C : Establishment of occupation chronicles in Grotte Mandrin using sooted concretions: rethinking the Middle to Upper Paleolithic transition. *J Hum Evol.* 2017;112:70–78. 10.1016/j.jhevol.2017.07.016 29037417

[ref-74] Vandevelde S Genty D Brochier JÉ : Des concrétions fuligineuses en contextes archéologiques: quel potentiel informatif? *Géomorphologie: relief, processus, environnement.* 2020;26(4):241–254. 10.4000/geomorphologie.14981

[ref-72] Zapata L Peña-Chocarro L : Uso y gestión del bosque en la Euskal Herria Atlántica: aprovechamiento tradicional de los recursos forestales en Encartaciones y Gorbea. *Zainak.* 2003;22:155–169. Reference Source

[ref-73] Ziemann MA Madariaga JM : Applications of Raman spectroscopy in art and archaeology. *J Raman Spectrosc.* 2021;52(1):8–14. 10.1002/jrs.6054

