# The “Archeology of the Light”:  A multiproxy, interdisciplinary and experimental approach to Paleolithic subterranean activities.

**DOI:** 10.12688/openreseurope.17712.2

**Published:** 2025-04-24

**Authors:** Mª Ángeles Medina-Alcaide

**Affiliations:** 1 PACEA-UMR CNRS 5199, Bâtiment B2 Allée Geoffroy Saint Hilaire, Universite de Bordeaux, Pessac CEDEX, 3615, France; 2 Universidad de Córdoba, UCO-Prehistoria, HUM-781, Córdoba, 14071, Spain

**Keywords:** Lychnology, Pyroarchaeology, Paleolithic Cave, Paleolithic Art, Internal Archaeological Context, charcoal, soot.

## Abstract

**Background:**

This article presents the A-LIGHT project and its main results. The "Archeology of the Light" (A-LIGHT) project aims to improve our knowledge of Palaeolithic cave activities through an interdisciplinary methodology applied to rarely-studied remains: the residues of Palaeolithic light from lamps, fireplaces and torches (specially, charcoal and soot).

**Methods:**

The methodology includes different stages such as: 1. Research in caves and sampling, 2 Laboratory analyses (multi-analytical approach adapted to the type of combustion residue analysed, including Anthracology, C14 dating, Bayesian analysis, SEM-EDX, TEM.EDX, Raman), 3. Ethnographic review of firelight, 4. Experimental reproduction and monitoring of Palaeolithic firelight. 5. Analyses, integration of data and synthesis.

**Results:**

This approach contributes multifaceted data about the Palaeolithic activities inside the caves (lighting systems selected, fuel used, chronology and intensity of visits or paleo-paths). Experimental reproductions have enabled evaluation of the Palaeolithic lighting potential. This provide essential information for research the visibility and the accessibility of Rock Art from GIS, and allow to more realistic virtual simulations.

**Conclusions:**

These data demonstrate that the *Archaeology of the Light* is “here to stay” and that it is an essential approach for a holistic understanding of Palaeolithic caves. Especially on the lighting systems used by paleo-groups in the underground environment (functioning, selected fuels, duration, light intensity), on the minimum number and date of prehistoric incursions, as well as on aspects related to the visibility and accessibility of Palaeolithic cave art.

## Introduction

*The earliest evidence* of “palaeospeleology” in Europe -meaning the presence of ancient humans in deep areas of the caves (in total darkness)-, has been linked to Neanderthals. This evidence discovered in Bruniquel Cave (France) and dated 176 ka BP, and comprised six circular anthropic structures, made up of approximately 400 fractured speleothems, with more than 18 fire traces inside, 336 m. from the entrance ( Jaubert *et al.*, 2016a). There is well-established evidence that hominins have been occupying caves for well over one million years ago, for example at Wonderwerk Cave in South Africa ( Chazan & Horwitz, 2023), among others. In this project, I limit the references about hominid cave use to the spaces with total darkness in caves and with fire evidences. In these deep areas the remains of fire undeniably come from the illumination use, without excluding other purposes, which allows to address the theme of the "Archaeology of Light".

There are other proposals regarding the beginning of human visits to deep underground environments, including some with chronologies older than Bruniquel, such as the Sima de Los Huesos in Cueva Mayor de Atapuerca (Spain, approximately 430 ka.: Aramburu *et al.*, 2017), the Dinaledi Chamber in Rising Star Cave (South Africa, between 241 and 335 ka: Dirks *et al.*, 2015), or Regourdou (France, 90 ka: Maureille *et al.*, 2015). However, so far, no evidence of lighting or fire has been found in them, an essential resource to confirm the intentionality of the visit and rule out a geological or transported origin of the remains.

However, in 2022, L. Berger (director of Rising Star cave) announced the discovery of combustion residues in the cavity (charcoal), which suggests the use of fire in an underground context by *Homo naledi*. However, to date, no scientific data has been presented to support this claim (for example, radiocarbon dating - which would exceed the instrumental limit for this method - or geoarchaeological characterisation of the context of these findings). Berger also proposed the existence of cave art and burials in the cave ( Berger *et al.*, 2023), issues that have also generated controversy and/or on which there is no generalized consensus ( Martinón-Torres *et al.*, 2024)

In the Upper Palaeolithic, combustion residues related to lighting proliferated in the inner spaces of caves, especially associated with graphic activity. However, archaeological research on these sites has traditionally focused only on Palaeolithic Art. Analysis of the Internal-Archaeological-Context began in the second half of the 20th century ( Clottes, 1993; Clottes & Simonnet, 1990). According to Jean Clottes (1993), this term refers to " *the remains and traces left by the activities of humans and animals in caves*", including the remains of fire and lighting. This internal context is now receiving increasing attention and is beginning to be approached in a holistic and interdisciplinary way in order to obtain global knowledge of the Palaeolithic anthropization of caves ( Arias & Ontañón, 2020; Garate, 2017; Jaubert *et al*., 2016b; Medina-Alcaide *et al*., 2018; Pastoors *et al*., 2017).

The main references on Palaeolithic lighting were published during the 1960s–1980s ( Allain, 1965; Beaune, 1987; Delluc & Delluc, 1979). These studies have mainly focused on a single type of lighting resource: portable lamps ignited with animal fat. Remarkably, this light-tool is the least frequent in the archaeological record and the one with the least light capacity. However, stone lamps were the only clear and archaeologically visible evidence at the time to study lighting technologies, particularly given the poor resolution of ancient cave excavations which likely did not record charcoals or other combustion remains. It is only recently that new excavations and advancements in methods have allowed for the study of heterogeneous combustion residues. Furthermore, these early studies were conducted at a time when physico-chemical analyses were only incipient within Archaeology. Now, there is an ideal research context to advance on this subject, even using portable and non-invasive tools ( Carrasco *et al*., 2016; Donais & Vandenabeele, 2021; Hernanz, 2015; Ziemann & Madariaga, 2021).

Charcoals, linked to the use of torches and firelight, are the most recurrent archaeological remains of light in the Internal-Archaeological-Context of Palaeolithic Art caves ( Medina-Alcaide *et al.*, 2018). Little attention has been paid to them, however, except in a few caves with exceptional conservation conditions ( Théry-Parisot & Thiébault, 2005; Théry-Parisot *et al*., 2018). This article presents the project *'The* " *Archaeology of Light": a multiproxy, interdisciplinary and experimental approach to* underground activities in the Palaeolithic', which places these remains (among other combustion remains) at the centre of research into knowledge of Palaeolithic underground anthropisation. In this article I present the A-LIGHT project (materials, approach and methodology) and its main results, some of which have already been published in different articles (Extended Data S.I.1). This article aims to provide an overview and summary of the discipline, as well as offer some new data and supplementary information.

## Methods

**Figure 1.**
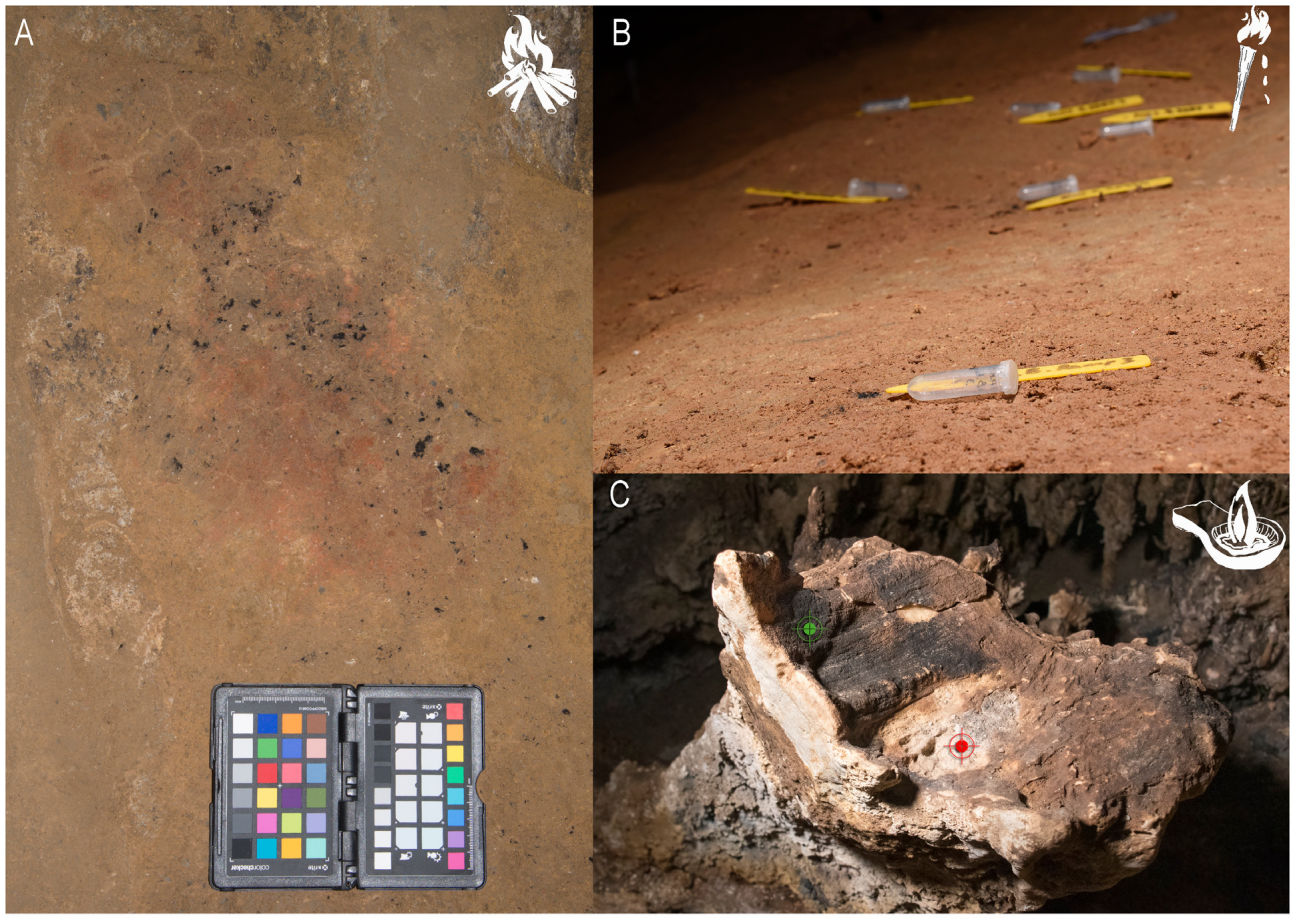
Diverse residues of Palaeolithic light systems analyzed in this project.

The main aim of this project has been to improve our knowledge of the early subterranean behaviour of Humanity, through a pioneering and interdisciplinary methodology applied on seldom studied remains: **the residues of Palaeolithic light, mainly charcoal and soot from fireplaces** ( Figure 1a: *fireplace from Atxurra with remains of charcoal, ash and rubefacted clay* – Garate *et al*., 2023), **torches** ( Figure 1b: *scattered charcoal linked to the use of wooden torches from Alkerdi 2* cave) and **lamps** ( Figure 1c: *fixed “lamp” of Nerja cave with soot deposit -green- and micro-charcoal remains -red*). For more information on the type of residues generated by each lighting system, as well as on its different materialities in endokarst, see Medina-Alcaide *et al.*, 2021.

**Figure 2.**
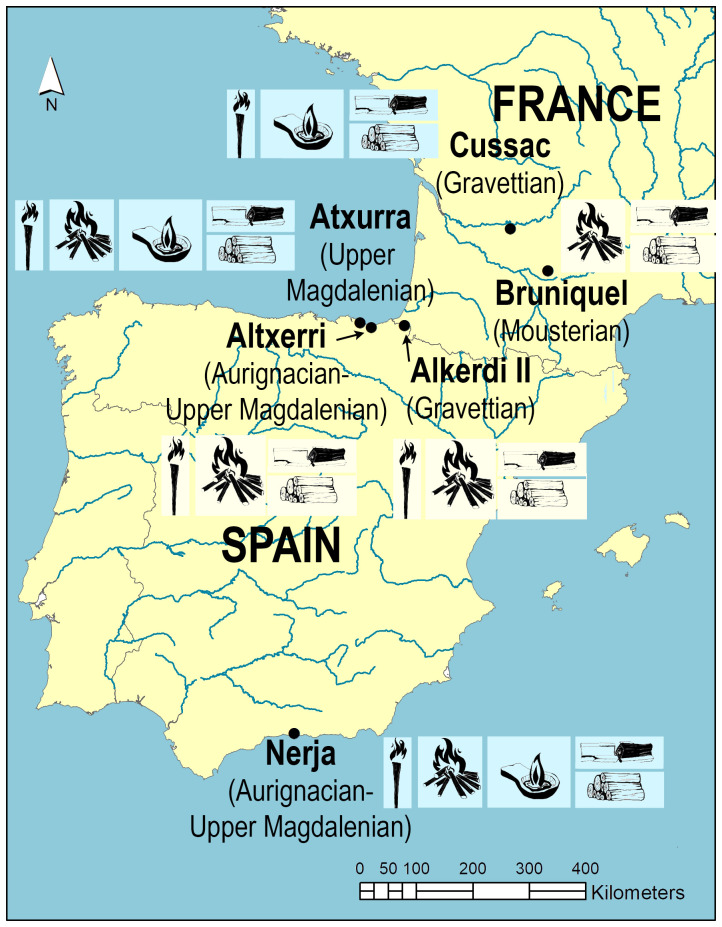
Site location, chronology, types of lighting and fuel.

This research focuses on outstanding remains of Palaeolithic lighting from **six European caves** ^1^, one of which has been declared a World Heritage Site by UNESCO (Altxerri cave) ( Figure 2). These remains were carefully selected in order to: (1) analyse and compare the different lighting systems, (2) extend the analysis to combustion remains of various types (charcoal and soot), (3) expand the archaeological record studied at the international level (France and Spain) and (4) extend the period studied, while incorporating the knowledge of lighting technology possessed by different Hominin (Neanderthal and *H. sapiens*). Specifically, the inner anthropization of the selected sites integrates different phases of the Upper Palaeolithic (35–12 ka. BP), including a period of the Middle Palaeolithic (176 ka. BP). This contributes to a comprehensive diachronic understanding of the topic. Although the project includes six caves, this article mainly presents results from Nerja and Atxurra caves, where I have obtained the most relevant results. The combustion residues from the other caves are still being analysed as part of other current research projects. 

The **objectives** of the project have been as follows: 1. Define and analyse the lighting systems used by palaeolithic hunter-gatherers in each cave, through multiproxy and multianalytical analysis (prioritizing on-site and non-destructive analysis). 2. Quantify the physical parameters and elemental characteristics, including their residual aspects, through experimental replication and monitoring. 3. Synthesize the data and evaluate the role of firelight in Palaeolithic hunter-gatherer societies, including its significance for the anthropization of caves and the performance of the first symbolic activities in them.

At the **methodological level**, the A-LIGHT project has comprised five Work Packages:

*1. Research in caves and sampling*: within the framework of the research projects in each cave, a systematic surface survey of each cave was carried out, as well as small probes in particular areas to assess the remains of anthropic activity underground beyond the surface level ( Medina-Alcaide *et al*., 2018). Dino-lite® portable microscopes (models AM4815ZT 20–200x, AM4013MZT4 400–470x, AM4515T8 700–900x) were used to conduct a preliminary characterization of the vestiges in caves, and Disto Leica (model X2 modified) for the topographical geolocalization on the 3D or the 2D topography of the caves ( Intxaurbe *et al*., 2020; Trimmis, 2018). The intrinsic and extrinsic characteristics of each combustion residue were recorded for the study. Sampling was carried out as aseptically as possible to avoid contaminating the sample for dating purposes, and it was done either manually for the remains on the surface or using a flotation system for the remains from the probes.

*2. Laboratory analyses*: On the charcoals, holistic anthracological analysis was implemented, covering, if possible, a taxonomic approach (to characterize the woody species of origin), a taphonomic approach (to identify the physiological and phenological state of the wood) and a dendro-anthracological approach (to determine the thickness of the wood). For this I used a light microscope (Leica DM2500M) and scanning electron microscope (JEOL IT 500 HR with EDS Oxford Instruments Ultim Max 100). This research also includes information on TEM-EDX spectroscopy (FEI Talos F200X equipment) proved extremely useful to characterize the prehistoric soot remains. 14C-AMS and UTh dating was used to determine the date of endokarst human use. For more information on chronometric techniques and the instrumentation used, see the specific article on this subject about Nerja cave ( Medina-Alcaide *et al.*, 2023).

Other specialists have collaborate actively in this methodological phase, for example, for the fuliginochronological analysis (S. Vandevelde, Université du Québec), for Raman analysis (D. Deldicque, École Normale Supérieure), for micromorphology analysis, to identify the ash and rubefaction clay, and various phases of visits inside the caves (C. Ferrier, Université de Bordeaux and M. Arriolabengoa, University of the Basque Country). During the years of the project, I have published various work with them, where additional data can be consulted on the methodology and specific techniques used for the interdisciplinary study of combustion residues (Extended Data S.I.1, Deldicque *et al.*, 2023; Garate *et al.*, 2023; Medina-Alcaide *et al.*, 2023).

3. Ethnographical review of firelight: This project includes a review of the ethnographic literature on fire lighting in circumpolar societies (Evenki, Inuit), frequently compared in the literature with Palaeolithic groups due to their lifestyles, and on the last human groups that lived in caves (the Tao't Bato tribe). However, this aspect has not been developed in depth. It will be addressed in future researches. This section will not be approached through direct parallels, only as a means of reflection and to open up horizons of aspects that are difficult to trace through the archaeological record, such as data on the system of mounting torches and other lighting systems or, on the social and cultural significance of firelight beyond practical activity.

4. Reproduction and monitorization of Paleolithic light: The experimental replication in an underground context under controlled conditions (monitoring and repeatability) of each type of recorded lighting system allowed us to quantify the physical parameters of Palaeolithic light. In particular, the lighting systems (fireplaces, wooden torches) found inside Atxurra cave have been replied. A possible lamp has also been located in this cave, but in the absence of information on how it worked, I reproduced a better-known example, the lamp from La Mouthe cave. These experimental activities were carried out in a natural cave, without archaeological remains, located in Lekeitio (Bizkaia, Spain). The cave has the necessary equipment to carry out the experimentation and monitoring of the light. In particular, I used a luxmeter (PCE-L335) and an infrared thermometer (model PCE-778) to monitor the fires, and OBO © Pro V2 sensors were also used to record the temperature, CO2 and humidity of the cave. For more information on the experimental aspect of the project, see the specific article on this topic ( Medina-Alcaide *et al.*, 2021).

In the final phase of the project, I also carried out experimental activities to quantify the waste produced by the wooden torches and to characterize the dispersion of these residues. This work has been carried out in collaboration with Gaëlle Rousseau, as part of her master’s degree project (“Archéologie Science pour l’Archéologie” master, University of Bordeaux 2024). This data is currently being analyzed and will be presented at the next GMPCA conference (2025).

5. Analyses, integration of data and synthesis: The R statistical package for statistical computation and graphics was used to explore the data set, as well as a heatmap tool in GIS to create a Kernel Density Map through vector point layers with the location of the lighting traces (archaeological and experimental residues), to observe the different distribution patterns and their relationship with other underground elements (structures or rock art,) and with different human movements inside the cave. The quantitative data obtained on Palaeolithic light (anterior section) has been used to develop GIS scripts to quantify the visibility and difficulty of access to deep areas with Palaeolithic Art. For more information on this aspect, see the published articles with Intxaurbe *et al.*, 2021; Intxaurbe *et al.*, 2022. The light parameters from the experimental tests were also used to develop virtual replicas of the one of the caves using © Unreal Engine 5 game engine. For more information, see the specific article ( Torres *et al.*, 2024). Besides, Bayesian analysis (©Oxcal 4.4.) was used for data management of the dating results (more information, in Medina-Alcaide *et al.*, 2023).

Finally, the results have been interpreted in the context of the available literature on Palaeolithic underground activities. The role of firelight in Palaeolithic underground behaviour was assessed based on the results obtained throughout the different methodological phases, with emphasis on the importance of light in the performance of the first symbolic activities in caves and on its economic and socio-cultural implications beyond purely utilitarian aspects.

## Results and discussion

In the following, I present the most outstanding results and interpretations obtained in the framework of the project "Archaeology of the Light" (A-LIGHT).

### Wood for light. Beyond a functional aspect?

**Figure 3.**
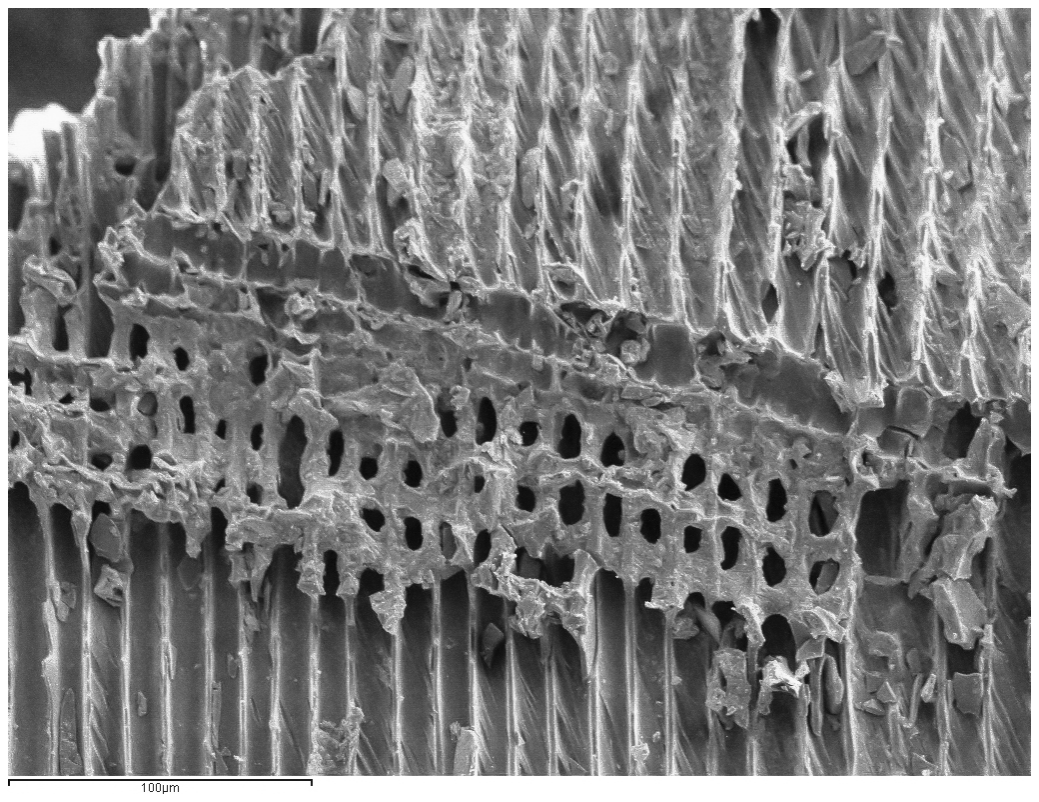
SEM photograph of *Pinus* tp. *sylvestris-nigra* charcoal from a Palaeolithic lamp.

Inside Nerja cave (Málaga, Spain), remains of three different lighting systems (torches, fires and fixed lamps) were found. The anthracological study of 336 charcoal from the woody fuel used in these prehistoric lighting resources shows a preference for the use of *Pinus sylvestris*- *nigra* (51.79%) ( Figure 3). Other taxa were also identified: *Pinus* cf. tp. *pinea*- *pinaster* (2.38%), *Pinus* sp. (3.87%), conifer (12.80%), Leguminosae (4.16%), Prunus sp. (0.30%), cf. Ulex sp. (0.30%), angiosperm (2.98%), indeterminable charcoals (7.44%) and pith remains (0.30%). In summary, I characterized 4 different types of taxa: *Pinus* tp. *sylvestris*- *nigra* (scots pine and black pine), *Pinus* cf. tp. *pinea*- *pinaster* (maritime pine and stone pine), *Prunus* sp. (blackthorn) and *Ulex* sp. (gorse). Other charcoal were defined with a lower degree of identification; which could correspond to the taxa mentioned above, or to others not identified at the taxonomic level. This was mainly due to the low consistency of most of the charcoals analyzed, which disintegrated when trying to prepare an optimal section for examination, and to the poor state of preservation of some of the remains. See Extended Data 2 (S.I. 2) for more information on the taxonomical analysis from Nerja caves charcoal. 

*Pinus* tp. *sylvestris*- *nigra* is the most common anthracological type found in the interior of European caves (France and Spain) and is linked to artificial lighting in the Upper Palaeolithic, specifically in the pre-Magdalenian period ( Medina-Alcaide *et al*., 2021). In some caves, it has even been documented almost exclusively: in Chauvet cave, for instance ( Théry-Parisot & Thiébault, 2005; Théry-Parisot *et al*., 2018), only one fragment of *Rhamnus* cf. (buckthorn family) was identified compared to 292 fragments of *Pinus sylvestris*. Environmental availability in the surroundings of the cave, the suitability of this wood for lighting the cave due to its resin content and other cultural aspects have been proposed as the reasons for this preferential choice ( Théry-Parisot & Thiébault, 2005; Théry-Parisot *et al*., 2018).

In Nerja cave all the identified woods used to light fires were also determined in fireplaces from habitat shelters, in the access areas of the cave ( Badal, 1996), as well as fitting in with other palaeoenvironmental studies in the south of the Iberian Peninsula, such as in the palaeopollen sequences from Bajondillo cave (Torremolinos, Málaga) ( López-Saez *et al*., 2007), from Gorham cave (Gibraltar) ( Carrión *et al*., 2008), from the Padul peat bog (Granada) ( Carrión, 2012; Pons & Reille, 1988), etc. In other words, the woody species used for illumination were **available in the cave environment** and do not seem to come from more distant areas. Specifically, all the radiocarbon dates that were carried out on *Pinus* tp. *sylvestris*- *nigra* charcoal have a Palaeolithic chronology (>15000 years ago), and in no case a Holocene chronology (more information, in Medina-Alcaide *et al.*, 2023). These palaeoenvironmental data are in line with the anthracological study carried out in the stratigraphic sequence of the Vestibule (external room of the cave) ( Badal, 1996), where, although this wood is present throughout the stratigraphic sequence from the Gravettian to the Epipalaeolithic, it loses its predominant character from the Upper Magdalenian onwards, and it is present in low percentages. The choice of *Pinus sylvestris-nigra* wood for illumination activities could be an intercultural aspect that has been suggested by other researchers. This has been pointed out for Chauvet cave where there is a main use of *Pinus sylvestris* as a lighting fuel during the Aurignacian and Gravettian periods ( Théry-Parisot *et al.*, 2018). In Nerja cave, I have recorded its preferential use from the Aurignacian to the Upper Magdalenian, and therefore the anthracological data from this project reinforce this idea of a cross-cultural choice, although other woods were available in the environment ( Badal, 1996). This choice has been recorded even at times when the presence of this type of pine is in decline (Upper Magdalenian).

The anthracological results also agree with the socio-economic interpretation proposed in the previous anthracological study of Nerja cave ( Badal, 1996), regarding the avoidance of stone pine wood as fuel, due to the scarce fragments of this carbonized wood recognised throughout the external stratigraphic sequence and the abundance of pine cone bracts and pine nut shells from this tree. This led Badal and colleagues to consider the exploitation of this tree for food rather than fuel ( Badal, 1996; Badal *et al*., 2014). The new data confirm this management of vegetable resources by Palaeolithic groups and the prior **planning of wood used** as fuel depending on the activity. Specifically, the fuel used for lighting is mainly *Pinus* tp. *sylvestris*- *nigra*, with *Pinus* tp. *pinea*- *pinaster* being very sparsely represented (2.38%).

The manual collection of the remains could also be one of the causes of the overestimation of some taxa over others ( Chabal, 1997). In fact, when I applied a more exhaustive collection of the carbonized remains (by flotation), for example in the case of the Ledge of Horses in the Atxurra cave ( Garate *et al*., 2023), a greater number of taxa were found. For more information, see the specific article where we published this data ( Garate *et al.*, 2023). The variability of taxa is not high, however, and the representativeness of some taxa over others continues to be eloquent in favour of one or, at most, two determinations. In this sense, ethnographic studies show that monofunctional fires, i.e. those linked to a specific activity (such as lighting in our case), have a higher degree of fuel selection ( Henry *et al*., 2018).

Concerning the taphonomic alterations of the analysed charcoals, I observed different anomalies linked to the combustion process, such as vitrification (54.14%) and shrinkage cracks (32.41%). See Extended Data (S.I. 3) for more information on the taphonomic analysis of the Nerja cave charcoals. Vitrification is related to several factors, some of which may fit with the particularities of the context of origin of our charcoals, for example: a. the sudden interruption of combustion as a trigger for vitrification ( Carrión, 2005) could be connected, in my context, to the gradual detachment of the charcoal from the torches (during the pyrolysis stage); b. the burning of **small branches** ( Marguerie & Hunot, 2007) could correspond to the diameter of wood suitable for transport to deep contexts of the caves, as well as its suitability for the manufacture of torches; c. the burning of dense wood with high **resin content** ( Py-Saragaglia & Ancel, 2006; Scheel-Ybert, 1998) could be linked with the taxonomic identification of most of our charcoal samples (pine and conifer wood); d. the aerobic degradation of the wood prior to combustion ( Henry, 2011) could be linked to the **use of dead wood** derived from natural pruning for lighting ( Théry-Parisot & Thiébault, 2005; Théry-Parisot *et al*., 2018). Concerning the last point, the identification of several samples with a strongly altered structure (44.14%) and with low consistency (hyperfragmentation) could also indicate a preferential use of dead wood, which is easier to collect and ignite, for artificial lighting ( Asouti & Austin, 2005; Scheel-Ybert, 2001; Théry-Parisot, 2001). In addition, this preference would imply a strong **seasonal component for the development of subterranean activity**, outside the snow season, **or planning of this activity** by taking into account the necessary drying time of the wood (at least two years) ( Théry-Parisot *et al*., 2018). Only dried wood guarantees a homogeneous and persistent combustion of flammable gases. However, in the experimental activities, good results were obtained with a slightly shorter dehydration time in a dry and sheltered environment.

**Figure 4.**
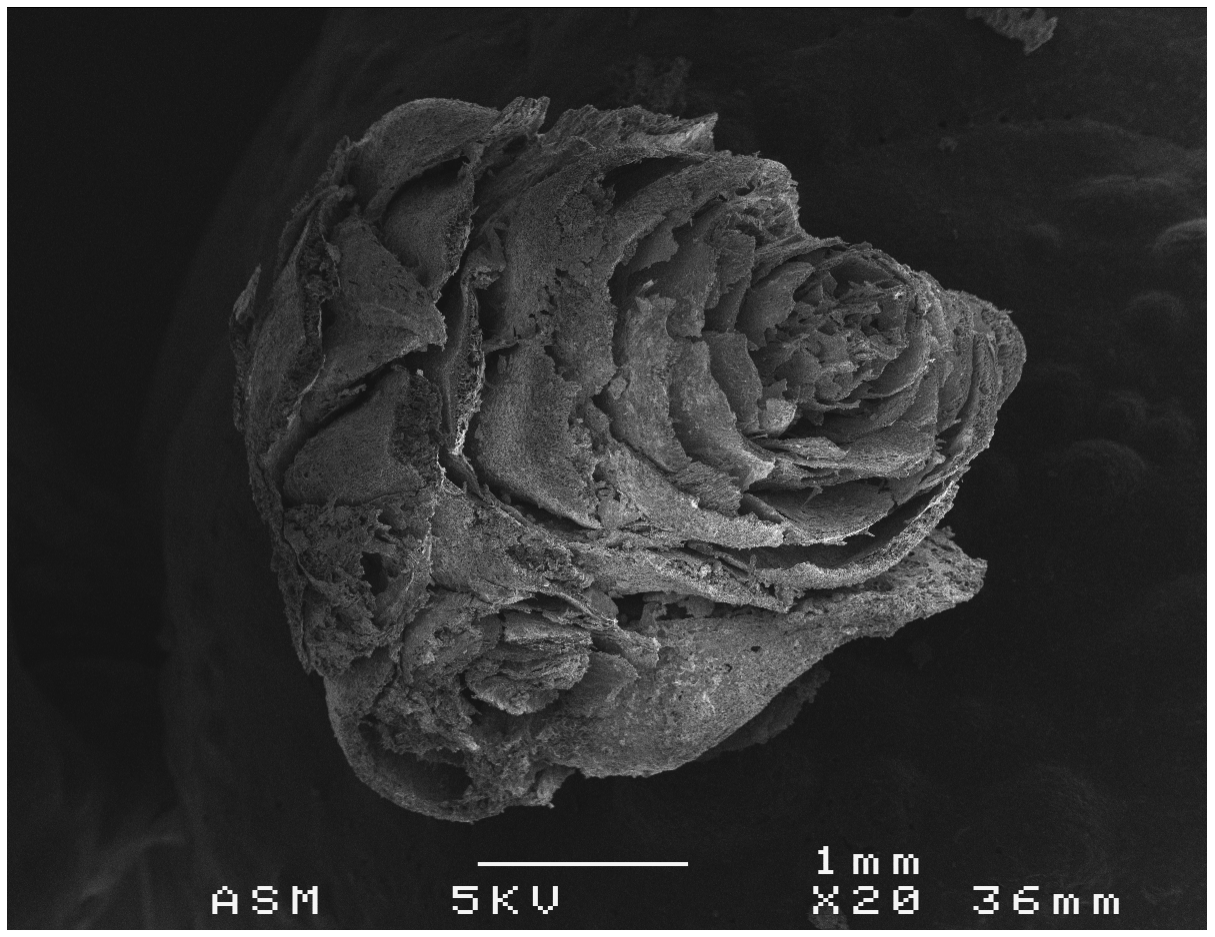
SEM photograph of *Pinus* sylvestris buds.

Also related to this seasonal aspect, I must highlight the vegetative bud of *Pinus sylvestris* found in the internal context of the Nerja cave and related to Gravettian charcoal (27000 years ago) linked to the use of wooden torches ( Figure 4) ( Medina-Alcaide *et al*., 2015). This element is unique in similar contexts. These slightly resinous buds develop at the tips of branches during autumn and winter ( Ruiz de la Torre, 2006). If the wood had been harvested when green, I could infer some seasonal data about the time of the year when the visit may have taken place; however, the data obtained to date suggest the harvesting of dead or dry wood, so these shoots may have been part of the fuel collected after natural pruning. However, the discovery of this remain indicates that **the fixed fires or torches could include other plant elements** apart from woody fuel, such as this resin-rich shoot.

Finally, the charcoals examined show brownish stains (22.07%). Based on experimental observations, I attribute these stains to the partial and incomplete burning of the wood. This likely occurred when charcoal fell from the torches before complete combustion; some remains could also be the result of partial combustion of a fixed fire.

In addition, traces of reaction wood were identified ( Figure 3) (31.72%), together with some knots (1.38%). These alterations suggest that the firewood used must have come from a group of **thin branches**. In addition, dendro-anthracological analysis has been carried out on some of these coals (very few remains, as most of the coals were very small and fragile). Of the remains analysed in Nerja cave, only 9 fragments were found to have moderate ring curvature, 21 had strongly curved log rings and the vast majority (259) were classified as having indeterminate ring curvature. This was determined by observing the degree of curvature of the rings in the cross-section of the charcoal ( Marguerie & Hunot, 2007). In this sense, it can be deduced that small branches were preferably used. However, none of the charred wood remains preserves the bark, so we cannot know if the charcoal fragment corresponds to small branches or to portions close to the pith. In any case, the context of the origin of the fires, the deep and rugged areas of the cave, limits the use of large logs, as well as the very manufacture of the torch for optimal performance. In addition, previous studies proposed the choice of small branches for lighting caves based on the size of the wood remains preserved in some caves (deep areas), for example, the traces of wood found in the clay on the floor of Chauvet cave ( Clottes, 2001), or the wooden branches preserved under the concretion in Aldène cave ( Galant *et al.*, 2007).

The presence of hyphae was also recorded (32.76%), preferably post-combustion (white). This anomaly is linked to the endokarst microbial activity and to the high humidity. As far as possible, I preferred not to send any charcoal with this type of alteration to be dated, as it has been related to the possible rejuvenation of some radiocarbon dates obtained in similar contexts (for example, in Tito Bustillo cave - Fortea, 2007).

In summary, beyond the species, the state or the size of the wood chosen as fuel, various ethnobotanical studies also show that historical and cultural conditioning factors influenced the selection of wood for firewood, even when other species were abundant in the vegetation ( Henry *et al*., 2009; Henry *et al*., 2018; Lavrillier, 2007; Zapata & Peña-Chocarro, 2003). At the same time, socio-economic factors have been pointed out in the choice of fuel, such as the previously cited example of Nerja Cave ( Badal, 1996; Badal *et al*., 2014). While the cultural and symbolic conditioning factors are difficult to ascertain from the archaeological record, they must nevertheless be taken into account, particularly in the archaeological context examined, which is often far removed from basic subsistence activities and subject to behaviours of a symbolic or ritual nature.

### Prehistoric cave life through soot and charcoal trails

Through the C14-AMS dating of 53 of these charcoals, a high-precision Bayesian model to identify the **minimum number of prehistoric visits** to the interior of Nerja cave has been constructed. For more information on the materials and the methodology used, see the specific article published within the project ( Medina-Alcaide *et al.*, 2023). The different phases were defined according to the presence of archaeological materials (and not only through the radiocarbon dating results) of each of the proposed phases in the stratigraphic deposits of the entrance rooms of the cave, or, failing that, to the presence of this type of cultural material in the regional context. For this purpose, using OxCal4.4, we sequenced these suggested Phases, limited by Boundaries determining the temporal distributions associated with the changes of phases. Outlier Charcoal model ( Bronk Ramsey, 2009) was used to properly down-weight outlier samples, taking into account the long life of the charcoal samples. This allowed, first, confirming the statistical plausibility of the model, then determining the start date (beginning of the boundary) and the end date (end of the boundary) for the different phases of human presence within the cave, as well as their duration and the transition period between phases ( Bronk Ramsey, 1995; Bronk Ramsey, 2001; Bronk Ramsey & Lee, 2013; Morrell, 2019).

**Figure 5.**
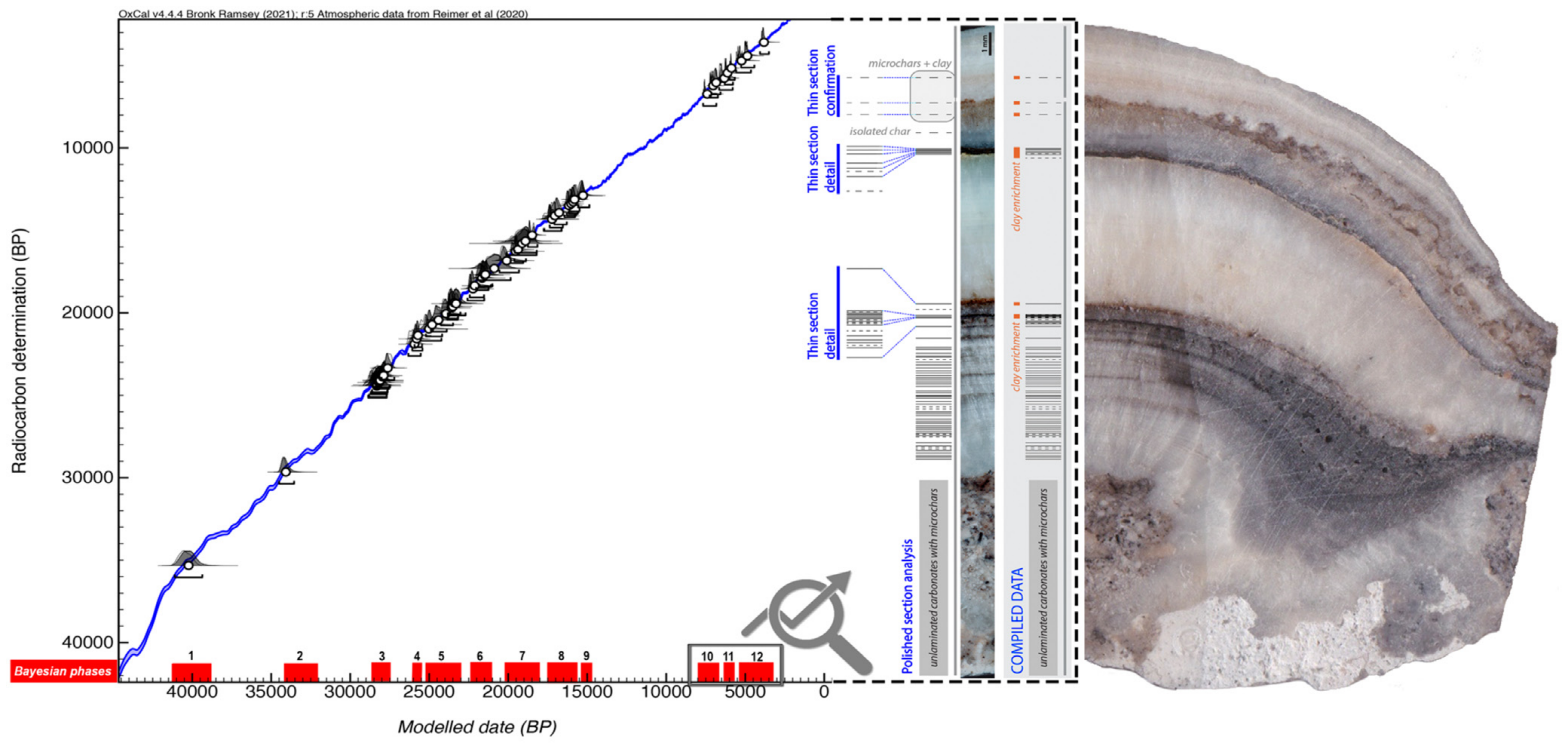
Phases of visits inside of Nerja cave identified by Bayesian model and micro-layers of soot.

The Bayesian model suggests at least **12 distinct phases** of visits to the interior of Nerja cave between **41,218 and 3299 cal BP**, with an agreement index (Aoverall) of 98. These phases of visits to the interior of the cave correspond to the specific chronocultural periods for the prehistoric regional context and with transitional periods: Early Aurignacian (phase 1), Recent Aurignacian (phase 2), Gravettian (phase 3), Lower Solutrean (phase 4), Middle Solutrean (phase 5), Upper Solutrean (phase 6), transition between the Upper Solutrean and Lower Magdalenian, and Lower Magdalenian (phase 7), Middle Magdalenian (phase 8), Upper Magdalenian (phase 9), Early Neolithic (phase 10), Recent Neolithic (phase 11) and Copper Age (phase 12) ( Figure 5).

There are 12 possible transition intervals between the different phases. One of them, separating phases 9–10 (between the Upper Magdalenian and the Early Neolithic), has a notable chronological amplitude (> 6000 years). There are however 8 transition periods that could correspond to 0 years ( **almost-continuous phases**, taking into account the minimum values), in particular, those between phases 1–2 (both could be included in the Aurignacian, the former probably belonging to the ancient phase and the latter to the evolved stage), 4–5 (between the Lower Solutrean and the Middle Solutrean), 5–6 (between the Solutrean and the Middle Solutrean), 6–7 (around the Upper Solutrean and the transition between the Upper Solutrean and Lower Magdalenian), 7–8 (between the Lower Magdalenian and Middle Magdalenian), 8–9 (between the Middle Magdalenian and Upper Magdalenian), 10–11 (between the Early Neolithic and Recent Neolithic), and 11–12 (between the Recent Neolithic and Chalcolithic).

Through the identification, microcounting and dating of micro-layers of soot (fulinochronological analysis - Vandevelde *et al*., 2017; Vandevelde *et al*., 2018; Vandevelde *et al*., 2020; Vandevelde, 2021) present inside a small stalagmite, the minimum number of visits for the last three phases identified in the Bayesian analysis was specified. This multianalytic approach was developed in collaboration with S. Vandevelde, E. Pons-Branchu and H. Valladas (Laboratoire des Sciences du Climat et de L’Environnement, LSCE/IPSL, CEA-CNRS-UVSQ, Université Paris-Saclay). The chronology of the soot microlayers found in the stalagmite was determined by the C14-AMS dating of CaCO3 layers deposited before and after them, following the methodology previously used in other studies on Nerja cave ( Pons-Branchu *et al*., 2022; Sanchidrián *et al*., 2017; Valladas *et al*., 2017). The soot deposits at the base of the stalagmite were deposited between 7562 and 6736 cal. BP; the soot microlayers from the upper level of the stalagmite were deposited between 6836 and 2998 cal. years BP assuming 0% of DCP for the calibration of the dating.

A minimum of **64 occupations were identified between 7000 and 3000 years** (a Minimum Number of Occupations—MNO that can be increased to 82 if uncertain soot films and microcharcoal alignments are also included; this second case will be indicated in brackets from now on). If we relate these data to the phases determined by Bayesian analysis, we can state that at least 58 (71) different occupations occurred in phases 10 and 11 (Early Neolithic and Recent Neolithic) with no apparent hiatus between these two phases (a result that is consistent with the Bayesian model from charcoal), and at least 6 (8) visits in phase 12 (Copper Age). The Figure 5 (modified from Medina-Alcaide, Medina-Alcaide *et al*., 2023) shows different phases of visits to the interior of Nerja cave identified by a Bayesian model from charcoal (in red) and an image of the different soot micro-layers of soot of the stalagmite, which increases the minimum number of occupations to at least 64 for the last 3 Bayesian phases. The succession of soot films in the carbonates is represented as barcode diagrams. Bars represent soot films and dashed lines represent probable soot films. The long vertical grey line next to the barcode represents speleothem total thickness.

**Figure 6.**
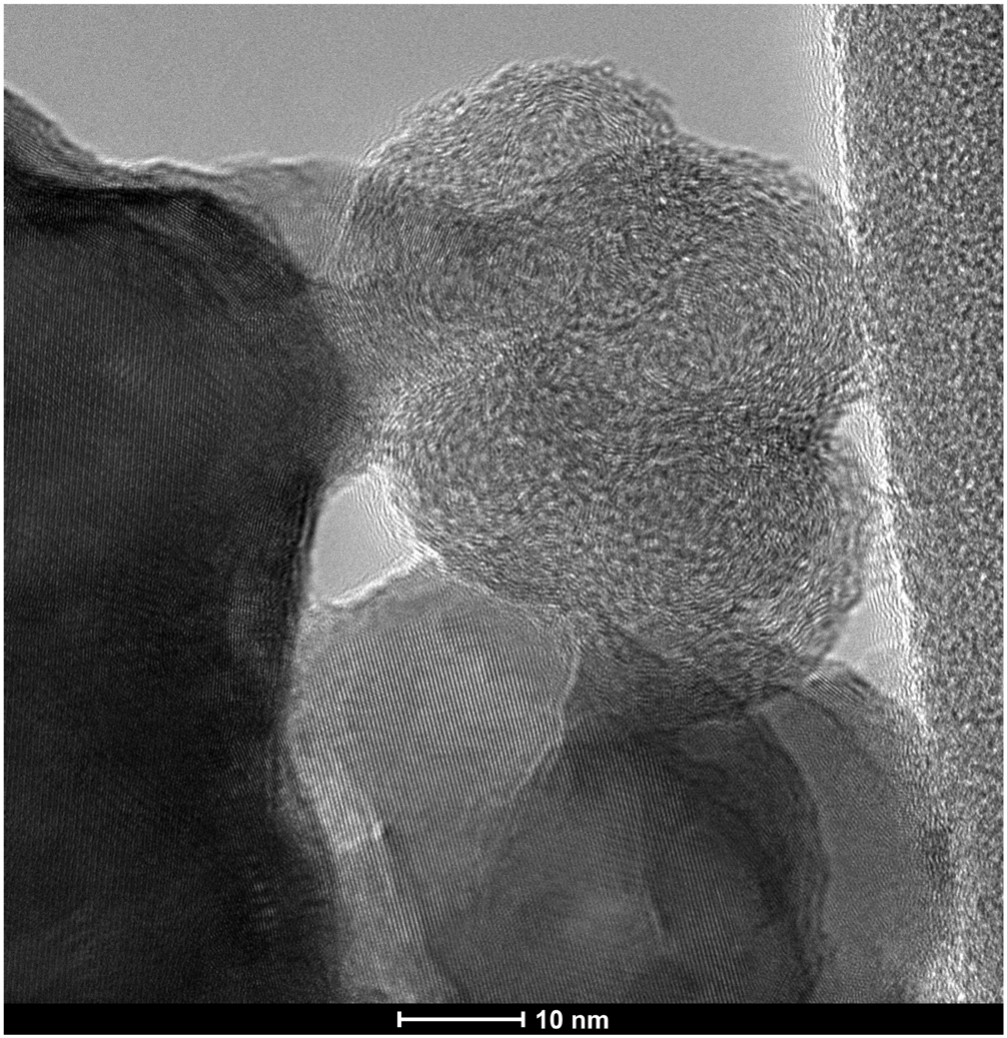
TEM-EDX photograph of nanometric spherical particles of prehistoric fossilized soot.

The identification of the soot remains inside the stalagmite as microlayers was characterised by TEM-EDX (following Pawlyta & Hercman, 2016) and Raman analysis (following Sadezky *et al*., 2005 and Deldicque *et al*., 2016). TEM–EDX observations revealed spherical carbon particles of soot aggregates ( Figure 6). In Nerja Cave, similar particles were observed inside a Palaeolithic fixed lamp in the upper galleries ( Medina-Alcaide *et al*., 2019), but soot residues were also located in another speleothem fragment inside the cave ( Del Rosal, 2016; Pons-Branchu *et al.*, 2022). Microscopic observation also revealed clay deposits together with microcharcoal and soot levels, suggesting that clay was not brought in by percolation at different stages of the stalagmite formation, but that the human visits to the cave contributed to the suspension of clays, which re-deposited in the stalagmite along with the particulates from wood combustion (soot and microcharcoal).

### Reliving Palaeolithic light for a better understanding of Palaeolithic cave art

**Figure 7.**
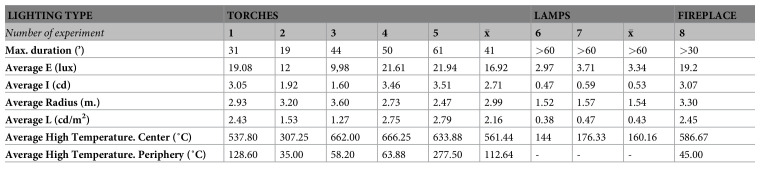
Average values of the parameters measured in each experiment with Palaeolithic torches, lamps and fireplaces.

**Figure 8.**
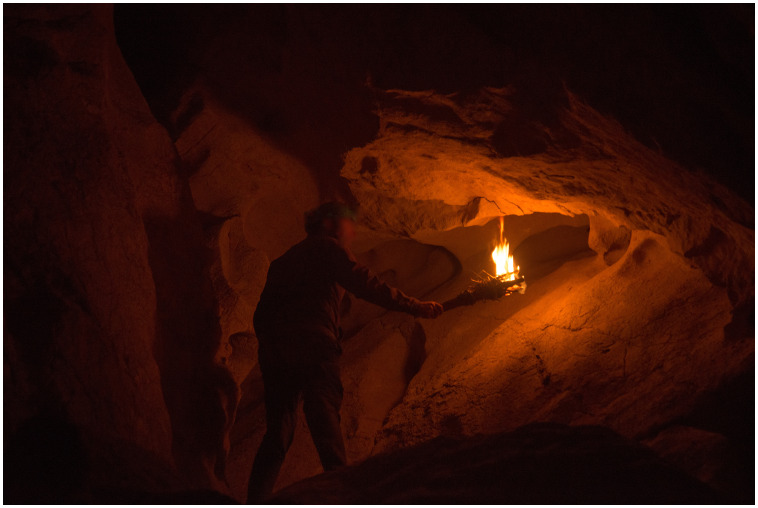
Photograph of the experimental recreation of Palaeolithic lighting with torches.

The **experimental replication of Palaeolithic light** was monitored in an endokarst environment in order to resemble the humidity, temperature and moisture conditions of the Palaeolithic context as closely as possible. For more information on experimental protocol used, see the specific article published within the project ( Medina-Alcaide *et al.*, 2021). The physical parameters obtained for the three Palaeolithic lighting systems are shown in Figure 7– Figure 8. These light resources were constructed on the basis of an exhaustive compilation of the existing literature on the subject, which also included archaeological data obtained.

The functioning of five wood torches was evaluated in a cave context (experiments 1–5 ( Figure 7). These torches were all made from branches of dry juniper wood 1.2 cm thick that were joined together. This type of structure was chosen because it fits with the archaeological data and the form of the few prehistoric torches that have been preserved. For example, it matches the thickness of the remains of branches that have been found in the caves of Aldène and Réseau Clastres and with the morphology of a Hallstatt torch ( Medina-Alcaide *et al*., 2021). Birch bark was included with the juniper branches as a form of tinder to start combustion. Similarly, the wood was broken up to facilitate combustion (through easier oxygenation) and impregnation with non-ligneous fuel. Thus, pine resin, animal fat, or a combination were added in some cases to assess the function of the torches with those fuel types.

For fireplaces, the wood fuel used was thin branches of juniper and oak wood in a dry state, arranged in a tepee-shaped structure. Birch bark was used to start the fire. This was placed inside the combustion structure. The fireplace was 23 cm in diameter and 7 cm high (measured before lighting), had no boundary structure, and was lit on a clay substrate. This experiment was carried out at a distance of 80 cm from the closest wall and 1.60 m from the ceiling in order to better assess the reflection of the light.

The lamps used in the experiments were replicas of the lamp from La Mouthe cave (Dordogne, France) ( Berthelot, 1901). This lamp was made of sandstone, 17 cm long and 12 cm wide, and had a concavity with ≈150 cm ^3^ capacity. Bovid marrow was used as the main fuel, with three woody wicks composed of dried and crushed juniper wood. They were arranged in a tepee shape in the center of the active part of the piece. Pine resin was added in one experiment (number 7) to assess its lighting benefits.

**Figure 9.**
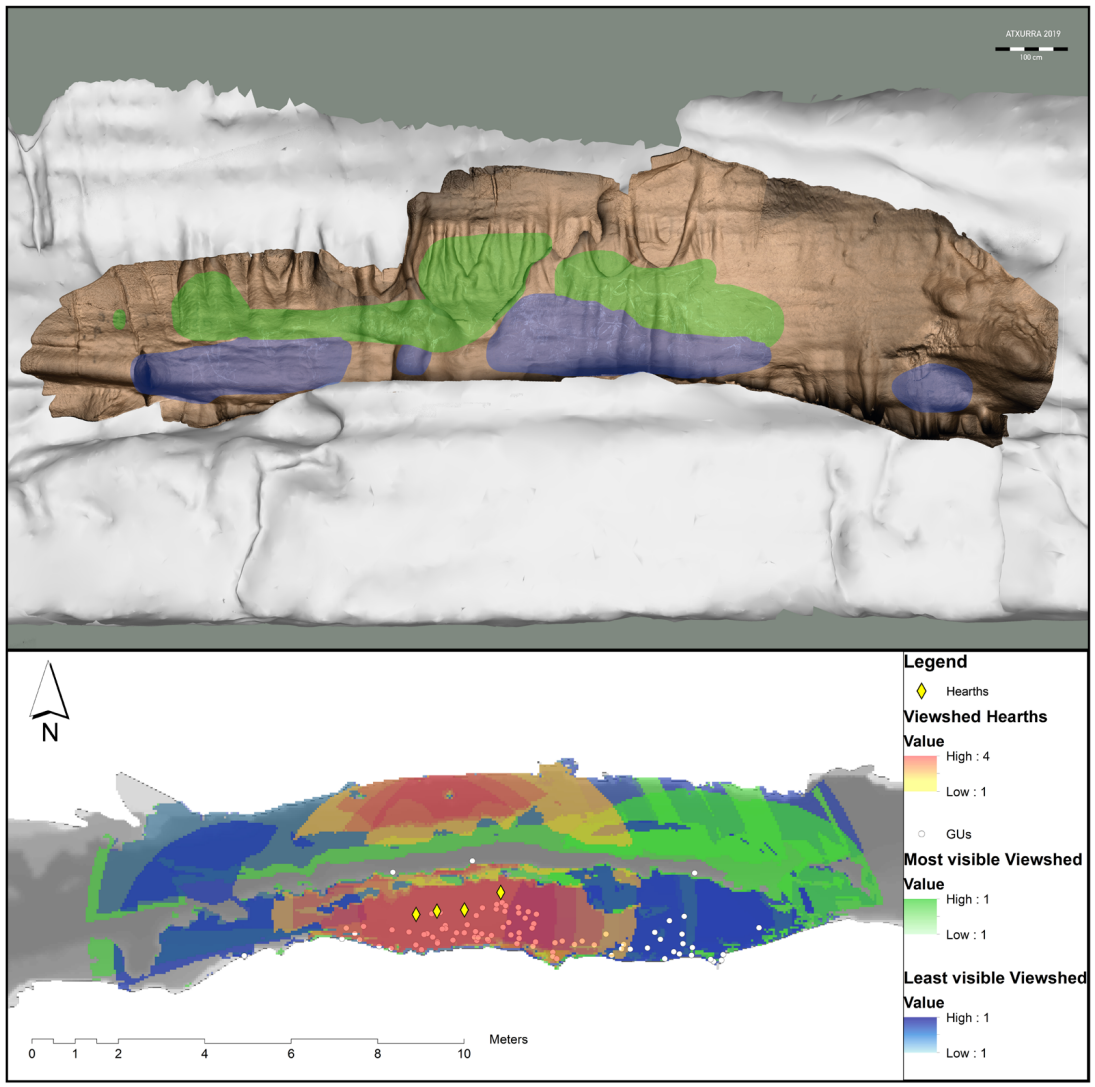
Viewshed analysis of Sector J of Atxurra cave.

The potential of GIS (ArcGIS), together with the physical parameters of Palaeolithic light, deduced in the experimental phase of this project, have made it possible to design a cutting-edge methodology to quantify visibility and capacity in sectors with Palaeolithic art inside caves (for more information see specific articles, Intxaurbe *et al.*, 2021; Intxaurbe *et al.*, 2022). Figure 9 shows the 3D restitution of the rock art combined with the 3D model of the cave ( Garate *et al.*, 2023) together with the visual basins of the Graphic Units in Sector J using ArcMap. It can be seen that the most visible figures are on a high panel. Furthermore, the estimation of the maximum potential audience for the Palaeolithic Art in this area of Atxurra cave and the optimal location for visibility were calculated, by creating a PYTHON script that was integrated into the software and provided a comparison with quantitative data between different caves. This innovative methodology was successfully applied in three renowned caves in the Basque Country: Santimamiñe, Altxerri and Atxurra. It showed that, in some cases, there may have been prior planning (specific location) to improve the visibility of some figures. In any cases, the groups of figures are found in deep and hidden parts of the caves, normally in sectors with a limited capacity to accommodate people, which would be consistent with a restricted communication system ( Intxaurbe *et al.*, 2021).

In the paradigmatic case of the shelf in sector J of Atxurra, **the location of the fireplaces** at the foot of the rock art ensemble (dated to the same period as the artworks themselves on the adjacent wall) **seems to have been determined to provide visibility**, as firelight halo would have illuminated almost the entire wall with Palaeolithic Art in this zone ( Garate *et al*., 2020a; Garate *et al*., 2020b; Medina-Alcaide *et al*., 2021), thus increasing the visibility indices of the figures on the right side of the wall. This is even more prominent for the two main horses of the composition, as they feature various techniques of execution such as engraving and scraping, and possibly painting.

Secondly, the physical parameters of Palaeolithic light obtained were used to propose a scoring methodology to quantify the accessibility of Palaeolithic Art inside the caves. The proposed methodology provides information on the difficulty of access, the estimated time of arrival, the length of the optimal route, etc. ( Intxaurbe *et al*., 2022). A PYTHON script was developed, integrating light and velocity data (experimental tests with professional cavers) for the spatial analysis of the 3D models of the caves through GIS (ArcGIS). This spatial study of the Palaeolithic Art in the caves makes it possible to compare the accessibility of the sites with quantitative (and not only qualitative) data, which is essential for investigating which graphical elements are more inaccessible or hidden and why.

This methodology was applied in Atxurra cave to evaluate the accessibility of all the rock art zones inside the caves. As a preliminary conclusion, the results obtained seem to show that the artists' choice of the panel was directly affected by the difficulty of transit (complex access via steep paths). The Upper Magdalenian groups at Atxurra chose panels that were difficult to access, despite the fact that the options offered by the cave are much wider and less risky. However, the reason for this is unknown: a desire to "hide" this art? An evidence of rites of passage? ( Arias, 2009; Owens & Hayden, 1997). In some cases, it seems that the locations in high areas could have been related to more functional reasons, such as giving **visual prominence to certain figures**, as in Sector J. This, as mentioned above, is accentuated by the location of the fires lit in this area of the cave. Logically, the estimated time to access each sector increases for areas that include constant vertical zones (passable by climbing or traversing). Experience, physical strength or knowledge of cave’s topography would help to optimize the time needed to access the deep areas ( Intxaurbe *et al.*, 2022).

**Figure 10.**
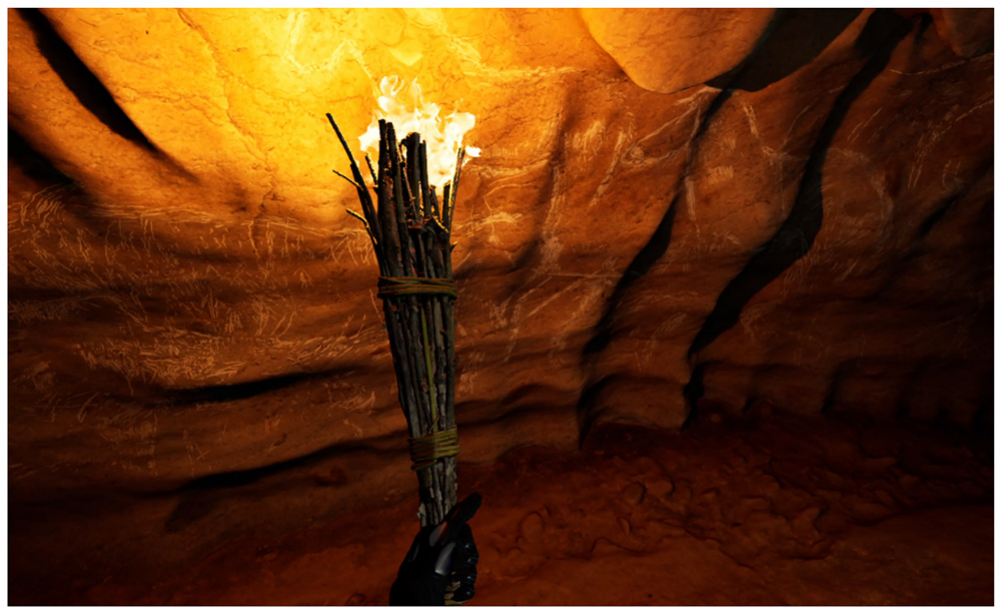
View of virtual reality of Atxurra rock art using torch lighting.

The light parameters from the experimental tests of the A-LIGHT project, in addition to the development of GIS analyses in caves, have also been used for the configuration of scientific virtual replicas. For more information, see the specific article ( Torres *et al.*, 2024). In this way, the data generated in the project has offered a resource that can potentially be transferred to society. This prototype has been developed in the © Unreal Engine 5 game engine, integrating the cave's 3D model, the rock art present in the Ledges of the Horses from the Atxurra cave, together with the two lighting systems located and studied through interdisciplinary analysis in this area: torches ( Figure 10) and fireplaces. Other outstanding studies on the virtualization of cave with Parietal Palaeolithic Art have used this data for specific analyses of lighting and visibility ( Wisher *et al.*, 2023; Wisher & Needham, 2023)

## Conclusion

The results obtained within the project entitled the A-Light reflect the potential of the interdisciplinary study of combustion and lighting remains in caves to enhance our knowledge of prehistoric subterranean activities, including rock art. The anthracological analysis of charcoal from illumination fires, including taxonomic, taphonomic and dendroanthracological approaches, suggests that there was a selection of fuel for cave lighting, probably linked to its environmental availability, its lighting capabilities and other economic and cultural reasons. Thanks to a multiproxy and interdisciplinary approach, the C14-AMS dating, the Bayesian analysis, and the interdisciplinary study of the soot deposits, the useful life of the cave (in the case of Nerja cave we have extended the origin of human occupation of this famous cave by 10000 years) and the minimum number of prehistoric visits to the cave interior have been defined. Experimental replication and monitoring of the physical parameters of Palaeolithic light in endokarst context has allowed for a better understanding of the functioning of these lighting systems, as well as the development of innovative methodologies for evaluating the visibility and accessibility of Palaeolithic Art inside the caves, together with virtual replicas of the underground environment with significant potential for the social and economic transfer of the data generated in the project. In short, these data demonstrate that the *Archaeology of the Light* is “here to stay” and that it is an essential approach for a holistic understanding of Palaeolithic caves.

## Ethics and consent

Ethical approval and written informed consent were not required.

## Data Availability

http://hdl.handle.net/10396/32709 No data are associated with this article No data are associated with this article http://hdl.handle.net/10396/32709

## References

[ref-1] Allain J : Les lampes magdaléniennes de Saint-Martin (Indre). In: *Congrès Préhistorique de France*.1965;178–183.

[ref-75] Aramburu A Arsuaga JL Sala N : The stratigraphy of the Sima de los Huesos (Atapuerca, Spain) and implications for the origin of the fossil hominin accumulation. *Quat Int.* 2017;433(Part A):5–21. 10.1016/j.quaint.2015.02.044

[ref-2] Arias P : Rites in the dark? An evaluation of the current evidence for ritual areas at Magdalenian cave sites. *World Archaeol.* 2009;41(2):262–294. 10.1080/00438240902843964

[ref-3] Arias P Ontañón R : La Garma: un sitio excepcional, una metodología diferente. In: *Actualidad de la investigación arqueológica en España I (2018-2019): conferencias impartidas en el Museo Arqueológico Nacional.*Subdirección General de Atención al Ciudadano, Documentación y Publicaciones, Madrid:2020;45–64. Reference Source

[ref-4] Asouti E Austin P : Reconstructing woodland vegetation and its exploitation by past societies, based on the analysis and interpretation of archaeological wood charcoal macro-remains. *Environ Archaeol.* 2005;10(1):1–18. 10.1179/env.2005.10.1.1

[ref-5] Badal E : La vegetation du Paleolithique superieur et de l’Épipaleolithique aux alentours de la Cueva de Nerja. *Actes du colloque de Périgueux 1995. Supplément à la Rev. d’Archéom.* 1996;171–176.

[ref-6] Badal E Vidal P Sanchidrián J : Pinions to eat? Again? *XVII World UISPP Congress.*Burgos.2014;256. Reference Source

[ref-7] Beaune SA : Lampes et godets au Paléolithique.Supplément à Gallia Préhistoire 23. Editions du Centre National de la Recherche Scientifique, Paris,1987. Reference Source

[ref-76] Berger LR Makhubela T Molopyane K : Evidence for deliberate burial of the dead by *Homo naledi*. *bioRxiv.* 2023. 10.1101/2023.06.01.543127 PMC12401548 40888484

[ref-8] Berthelot M : Sur une lampe préhistorique trouvé dans la grotte de La Mouthe. *Comptes Rendus Acad Sci.* 1901;666.

[ref-12] Bronk Ramsey C : Radiocarbon calibration and analysis of stratigraphy: the OxCal program. *Radiocarbon.* 1995;37(2):425–430. 10.1017/S0033822200030903

[ref-11] Bronk Ramsey C : Development of the radiocarbon calibration program. *Radiocarbon.* 2001;43(2A):355–363. 10.1017/S0033822200038212

[ref-10] Bronk Ramsey C : Dealing with outliers and offsets in radiocarbon dating. *Radiocarbon.* 2009;51(3):1023–1045. 10.1017/S0033822200034093

[ref-13] Bronk Ramsey C Lee S : Recent and planned developments of the program OxCal. *Radiocarbon.* 2013;55(2):720–730. 10.1017/S0033822200057878

[ref-14] Carrasco G Fortes-Román FJ Liñán-Baena C : Estudio de las alteraciones del soporte rocoso y de los espeleotemas de la Cueva de Nerja (Málaga) mediante tecnología LIBS. In: Andreo B, Durán JJ: (eds.): *El karst y el hombre: las cuevas como Patrimonio Mundial.*Asociación de Cuevas Turísticas Españolas. Madrid:2016;347–360.

[ref-17] Carrión JS : Paleoflora y paleovegetación de la Península Ibérica e Islas Baleares: Plioceno-Cuaternario.Ministerio Economía y Competitividad, Universidad de Murcia, Murcia,2012. Reference Source

[ref-16] Carrión JS Finlayson C Férnández S : A coastal reservoir of biodiversity for Upper Pleistocene human populations: palaeoecological investigations in Gorham’s Cave (Gibraltar) in the context of the Iberian Peninsula. *Quat Sci Rev.* 2008;27(23–24):2118–2135. 10.1016/j.quascirev.2008.08.016

[ref-15] Carrión Y : La vegetación mediterránea y atlántica de la península ibérica: nuevas secuencias antracológicas.Diputación provincial de Valencia, Valencia,2005. Reference Source

[ref-18] Chabal L : Forêts et sociétés en Languedoc (Néolithique final, Antiquité tardive): l'anthracologie, méthode et paléoécologie.Maison des Sciences de l'Homme, Paris,1997. 10.4000/books.editionsmsh.43380

[ref-77] Chazan M Horwitz LK : Wonderwerk cave and the kathu complex, South Africa.In: *Handbook of Pleistocene archaeology of Africa: hominin behavior, geography, and chronology.*Springer International Publishing, Cham,2023;1749–1765. 10.1007/978-3-031-20290-2_116

[ref-20] Clottes J : Contexte archéologique interne. In: G.R.A.P.P: (eds.): *L’art pariétal Paléolithique. Techniques et méthodes d’étude*. CTHS, Paris,1993;49–58.

[ref-21] Clottes J : La grotte chauvet: l'art des origines.Seuil, Paris,2001. Reference Source

[ref-19] Clottes J Simonnet R : Retour au Réseau Clastres (Niaux Ariege). *Préhistoire Ariégeoise.* 1990;45:51–139. Reference Source

[ref-25] Del Rosal Y : Análisis, impacto y evolución de biofilms fotosintéticos en espeleotemas. El caso de la Cueva de Nerja. PhD tesis. Universidad de Málaga,2016. Reference Source

[ref-23] Deldicque D Rouzaud JN Vandevelde S : Effects of oxidative weathering on Raman spectra of charcoal and bone chars: consequences in archaeology and paleothermometry. *C R Geosci.* 2023;355(G1):1–22. 10.5802/crgeos.186

[ref-22] Deldicque D Rouzaud JN Velde B : A Raman–HRTEM study of the carbonization of wood: a new Raman-based paleothermometer dedicated to archaeometry. *Carbon.* 2016;102:319–329. 10.1016/j.carbon.2016.02.042

[ref-24] Delluc B Delluc G : L´éclairage. In: Leroi-Gourhan A, Allain J. (eds.): *Lascaux inconnu.*Centre National de la Recherche Scientifique, Paris,1979;121–142.

[ref-78] Dirks PHG Berger LR Roberts EM : Geological and taphonomic context for the new hominin species *Homo naledi* from the Dinaledi Chamber, South Africa. *eLife.* 2015;4: e09561. 10.7554/eLife.09561 26354289 PMC4559842

[ref-26] Donais MK Vandenabeele P : Portable spectroscopy for on-site and *in situ* archaeology studies. *Portable Spectroscopy and Spectrometry.* 2021;1:523–544. 10.1002/9781119636489.ch44

[ref-27] Fortea J : 39 edades 14C AMS para el arte paleolítico rupestre en Asturias. *Excavaciones arqueológicas en Asturias, 1999-2002.* 2007;91–102. Reference Source

[ref-28] Galant P Ambert P Colomer A : Les vestiges d’éclairages préhistoriques de la galerie des Pas de la grotte d’Aldène (Cesseras Hérault). *Bulletin du Musée d'anthropologie préhistorique de Monaco.* 2007;47:37–80. Reference Source

[ref-29] Garate D : Más allá de la imagen: el arte parietal paleolítico en su contexto arqueológico. *Kobie Serie Anejo.* 2017;16:149–162.

[ref-30] Garate D Rivero O Rios-Garaizar J : The cave of Atxurra: a new major Magdalenian rock art sanctuary in Northern Spain. *J Archaeol Sci Rep.* 2020a;29: 102120. 10.1016/j.jasrep.2019.102120

[ref-31] Garate D Rivero O Rios-Garaizar J : Modelled clay animals in Aitzbitarte iv cave: a unique Palaeolithic rock art site in the Cantabrian Region. *J Archaeol Sci Report.* 2020b;31: 102270. 10.1016/j.jasrep.2020.102270

[ref-32] Garate D Rivero O Rios-Garaizar J : Unravelling the skills and motivations of Magdalenian artists in the depths of Atxurra cave (Northern Spain). *Sci Rep.* 2023;13(1): 17340. 10.1038/s41598-023-44520-w 37833336 PMC10575969

[ref-33] Henry A : Paléoenvironnements et gestion des combustibles au Mésolithique dans le sud de la France: anthracologie, ethnoarchéologie et exp ´erimentation. Tesis doctoral, Université Nice Sophia Antipolis, Nice,2011. Reference Source

[ref-34] Henry A Théry-Parisot I Voronkova E : La gestion du bois de feu en forêt boréale: problématique archéo-anthracologique et étude d'un cas ethnographique (Région de l'Amour, Sibérie).In: I. Théry-Parisot, S. Costamagno & A. Henry (eds.): *Gestion des combustibles au paléolithique et au mésolithique Nouveaux outils, nouvelles interpretations/Fuel Management during the Palaeolithic and Mesolithic Periods New tools, new interpretations.*BAR S1914, Oxford,2009;17–37. Reference Source

[ref-35] Henry A Zavadskaya E Alix C : Ethnoarchaeology of fuel use in northern forests: towards a better characterization of prehistoric fire-related activities. *Ethnoarchaeology.* 2018;10(2):99–120. 10.1080/19442890.2018.1510601

[ref-36] Hernanz A : Raman spectroscopy of prehistoric pictorial materials.In: P. Bueno-Ramírez & P.G. Bahn (eds.): *Prehistoric Art as Prehistoric Culture. Studies in Honour of Professor Rodrigo de Balbín-Behrmann.*Archeopress, Oxford,2015;11–20. Reference Source

[ref-37] Intxaurbe I Arriolabengoa M Medina-Alcaide MÁ : Quantifying accessibility to Palaeolithic rock art: methodological proposal for the study of human transit in Atxurra cave (Northern Spain). *J Archaeol Sci.* 2021;125: 105271. 10.1016/j.jas.2020.105271

[ref-38] Intxaurbe I Garate D Arriolabengoa M : Application of line of sight and potential audience analysis to unravel the spatial organization of Palaeolithic cave art. *J Archaeol Method Th.* 2022;29(4):1158–1189. 10.1007/s10816-022-09552-y

[ref-39] Intxaurbe I Rivero O Medina-Alcaide MÁ : Hidden images in Atxurra cave (Northern Spain): a new proposal for visibility analyses of Palaeolithic rock art in subterranean environments. *Quatern Int.* 2020;566–567:163–170. 10.1016/j.quaint.2020.04.027

[ref-41] Jaubert J Genty D Valladas H : The chronology of human and animal presence in the decorated and sepulchral cave of Cussac (France). *Quatern Int.* 2016b;432(Part B):5–24. 10.1016/j.quaint.2016.01.052

[ref-40] Jaubert J Verheyden S Genty D : Early Neanderthal constructions deep in Bruniquel cave in southwestern France. *Nature.* 2016a;534(7605):111–114. 10.1038/nature18291 27251286

[ref-42] Lavrillier A : Gestion duelle de l'espace à long terme chez les évenks éleveurs de rennes et chasseurs des Monts Stanovoï: interférences ou cohérences des zones sauvages et humanisées.In: S. Beyries y V. Vaté (eds.): *Les civilisations du renne d'hier et aujourd'hui. Approches ethnohistoriques, archéologiques et anthropologiques.*APDCA, Antibes,2007;65–88. Reference Source

[ref-43] López-Sáez JA López-García P Cortés M : Paleovegetación del Cuaternario reciente: estudio arqueopalinológico.In: M. Cortés (ed.): Cueva Bajondillo (Torremolinos). *Secuencia cronocultural y paleoambiental del Cuaternario reciente en la Bahıa de Málaga*. Diputación de Málaga, Málaga,2007;139–156. Reference Source

[ref-44] Marguerie D Hunot JY : Charcoal analysis and dendrology: data from archaeological sites in north-western France. *J Archaeol Sci.* 2007;34(9):1417–1433. 10.1016/j.jas.2006.10.032

[ref-79] Martinón-Torres M Garate D Herrie AIR : No scientific evidence that *Homo naledi* buried their dead and produced rock art. *J Hum Evol.* 2024;195: 103464. 10.1016/j.jhevol.2023.103464 37953122

[ref-80] Maureille B Holliday T Royer A : Importance of field data for understanding a potential Mousterian funerary deposit: the case of the Regourdou 1 skeleton (Montignac-sur-Vézère, Dordogne, France). *PALEO.* 2015;26:139–159. 10.4000/paleo.3678

[ref-47] Medina-Alcaide MÁ Cabalín LM Laserna J : Multianalytical and multiproxy approach to the characterization of a Paleolithic lamp. An example in Nerja cave (Southern of Iberian Peninsula). *J Archaeol Sci Report.* 2019;28: 102021. 10.1016/j.jasrep.2019.102021

[ref-49] Medina-Alcaide MÁ Garate D Intxaurbe I : The conquest of the dark spaces: an experimental approach to lighting systems in Paleolithic caves. *PLoS One.* 2021;16(6): e0250497. 10.1371/journal.pone.0250497 34133423 PMC8208548

[ref-48] Medina-Alcaide MÁ Garate-Maidagan D Ruiz-Redondo A : Beyond art: the internal archaeological context in Paleolithic decorated caves. *J Anthropol Archaeol.* 2018;49:114–128. 10.1016/j.jaa.2017.12.005

[ref-100] Medina-Alcaide MÁ Sanchidrián Torti JL Zapata Peña L : Lighting the dark: wood charcoal analysis from Cueva de Nerja (Málaga, Spain) as a tool to explore the context of Palaeolithic rock art. *Comptes Rendus Palevol.* 2015;14(5):411–422. 10.1016/j.crpv.2015.03.010

[ref-50] Medina-Alcaide MÁ Vandevelde S Quiles A : 35,000 years of recurrent visits inside Nerja cave (Andalusia, Spain) based on charcoals and soot micro-layers analyses. *Sci Rep.* 2023;13(1): 5901. 10.1038/s41598-023-32544-1 37041224 PMC10090096

[ref-51] Morrell B : La cronología como medio de interpretación social: los contextos funerarios del NE de la península ibérica entre finales del V inicios del IV milenio cal BC. Doctoral dissertation, Universitat Autònoma de Barcelona,2019. Reference Source

[ref-52] Owens DA Hayden B : Prehistoric rites of passage: a comparative study of transegalitarian hunter-gatherers. *J Anthropol Archaeol.* 1997;16(2):121–161. 10.1006/jaar.1997.0307

[ref-54] Pastoors A Lenssen-Erz T Ontañón R : With the back to the art. Context of Pleistocene cave art. *Quatern Int.* 2017;430(Part A):1–4. 10.1016/j.quaint.2017.03.033

[ref-55] Pawlyta M Hercman H : Transmission Electron Microscopy (TEM) as a tool for identification of combustion products: application to black layers in speleothems. *Ann Soc Geol Pol.* 2016;86(2):237–248. 10.14241/asgp.2016.004

[ref-56] Pons A Reille M : The holocene- and upper Pleistocene pollen record from Padul (Granada, Spain): a new study. *Palaeogeogr Palaeoclimatol Palaeoecol.* 1988;66(3–4):243–249, 255–263. 10.1016/0031-0182(88)90202-7

[ref-57] Pons-Branchu E Barbarand J Caffy I : U-series and radiocarbon cross dating of speleothems from Nerja Cave (Spain): evidence of open system behavior. Implication for the Spanish rock art chronology. *Quaternary Sci Rev.* 2022;290: 107634. 10.1016/j.quascirev.2022.107634

[ref-58] Py-Saragaglia V Ancel B : Archaeological experiments in fire-setting: protocol, fuel and anthracological approach. *BAR International Series S.* 2006;1483:71–82. Reference Source

[ref-59] Ruiz de la Torre J : Flora Mayor. Organismo Autónomo de Parques Nacionales, Madrid,2006. Reference Source

[ref-63] Sadezky A Muckenhuber H Grothe H : Raman microspectroscopy of soot and related carbonaceous materials: spectral analysis and structural information. *Carbon.* 2005;43(8):1731–1742. 10.1016/j.carbon.2005.02.018

[ref-60] Sanchidrián JL Valladas H Medina-Alcaide MÁ : New perspectives for ^14^C dating of parietal markings using CaCO _3_ thin layers: an example in Nerja cave (Spain). *J Archaeol Sci Rep.* 2017;12:74–80. 10.1016/j.jasrep.2017.01.028

[ref-61] Scheel-Ybert R : Stabilité de l’écosystème sur le litoral sud-est du Brésil à l’Holocène Supérieur (5500-1400 ans BP). Tesis doctoral, Université Montpellier II Sciences et Techniques du Languedoc, Montpellier,1998.

[ref-62] Scheel-Ybert R : Man and vegetation in southeastern Brazil during the late Holocene. *J Archaeol Sci.* 2001;28(5):471–480. 10.1006/jasc.2000.0577

[ref-66] Théry-Parisot I : Economie des combustibles au paléolithique: expérimentation, taphonomie, anthracologie. CNRS, Paris,2001. Reference Source

[ref-65] Théry-Parisot I Thiébault S : Le pin (Pinus sylvestris): préférence d'un taxon ou contrainte de l'environnement? Étude des charbons de bois de la grotte Chauvet. *Bull Soc Prehist Fr.* 2005;102(1):69–75. 10.3406/bspf.2005.13338

[ref-64] Théry-Parisot I Thiébault S Delannoy JJ : Illuminating the cave, drawing in black: wood charcoal analysis at Chauvet-Pont d'Arc. *Antiquity.* 2018;92(362):320–333. 10.15184/aqy.2017.222

[ref-85] Torres A Medina-Alcaide MA Intxaurbe I : Scientific Virtual Reality as a research tool in prehistoric archaeology: the case of Atxurra Cave (Northern Spain). *Virtual Archaeology Review.* 2024;15(31):1–15. 10.4995/var.2024.20976

[ref-67] Trimmis KP : Paperless mapping and cave archaeology: a review on the application of DistoX survey method in archaeological cave sites. *J Archaeol Sci Rep.* 2018;18:399–407. 10.1016/j.jasrep.2018.01.022

[ref-68] Valladas H Pons-Branchu E Dumoulin JP : U/Th and ^14^C crossdating of Parietal Calcite deposits: application to Nerja Cave (Andalusia, Spain) and future perspectives. *Radiocarbon.* 2017;59(6):1955–1967. 10.1017/RDC.2017.120

[ref-69] Vandevelde S : Les rythmicités d’occupation d’un site au sein d’un territoire. Approche diachronique.In: L. Slimak, Y. Giraud, L. Metz & P. Yvorra (eds.): *Mandrin. Des Derniers Néandertaliens Aux Premiers Hommes Modernes en France Méditerranéenne. A&t 4*. MMSH,2021;694–709. Reference Source

[ref-70] Vandevelde S Brochier JÉ Desachy B : Sooted concretions: a new micro-chronological tool for high temporal resolution archaeology. *Quatern Int.* 2018;474(Part B):103–118. 10.1016/j.quaint.2017.10.031

[ref-71] Vandevelde S Brochier JÉ Petit C : Establishment of occupation chronicles in Grotte Mandrin using sooted concretions: rethinking the Middle to Upper Paleolithic transition. *J Hum Evol.* 2017;112:70–78. 10.1016/j.jhevol.2017.07.016 29037417

[ref-74] Vandevelde S Genty D Brochier JÉ : Des concrétions fuligineuses en contextes archéologiques: quel potentiel informatif? *Géomorphologie: relief, processus, environnement.* 2020;26(4):241–254. 10.4000/geomorphologie.14981

[ref-81] Wisher I Needham A : Illuminating palaeolithic art using virtual reality: a new method for integrating dynamic firelight into interpretations of art production and use. *J Archaeol Sci Rep.* 2023;50: 104102. 10.1016/j.jasrep.2023.104102

[ref-82] Wisher I Pettitt P Kentridge R : The deep past in the virtual present: developing an interdisciplinary approach towards understanding the psychological foundations of Palaeolithic cave art. *Sci Rep.* 2023;13(1): 19009. 10.1038/s41598-023-46320-8 PMC10624876 37923922

[ref-72] Zapata L Peña-Chocarro L : Uso y gestión del bosque en la Euskal Herria Atlántica: aprovechamiento tradicional de los recursos forestales en Encartaciones y Gorbea. *Zainak.* 2003;22:155–169. Reference Source

[ref-73] Ziemann MA Madariaga JM : Applications of Raman spectroscopy in art and archaeology. *J Raman Spectrosc.* 2021;52(1):8–14. 10.1002/jrs.6054

