# Supplementary material for: The “Archeology of the Light”:  A multiproxy, interdisciplinary and experimental approach to Paleolithic subterranean activities
Source: Open Res Eur. 2025 Apr 24;4:216. Originally published 2024 Oct 14. [Version 2] doi: 10.12688/openreseurope.17712.2 (PMC12498324; doi:10.12688/openreseurope.17712.2)
Supplement: Peer review history

# Reviewer report: Reviewer response for version 2

**DOI:** 10.21956/openreseurope.21372.r53532

**Published:** 2025-04-30

**Authors:** Sally Hoare

**Affiliations:** 1 University of Liverpool, Liverpool, UK

Approved!

 The author has provided the required the changes.

If any, are all the source data and materials underlying the results available?

Partly

Is the work original in terms of material and argument?

Yes

Is the work clearly and cogently presented?

Partly

Is the argument persuasive and supported by evidence?

Partly

Does the research article contribute to the cultural, historical, social understanding of the field?

Yes

Does it sufficiently engage with relevant methodologies and secondary literature on the topic?

Yes

Reviewer Expertise:

Environmental archaeology, pyroarchaeology, fire and human evolution

I confirm that I have read this submission and believe that I have an appropriate level of expertise to confirm that it is of an acceptable scientific standard.

# Reviewer report: Reviewer response for version 1

**DOI:** 10.21956/openreseurope.19146.r48134

**Published:** 2025-01-17

**Authors:** Sally Hoare

**Affiliations:** 1 University of Liverpool, Liverpool, UK

In the article “Archeology of the Light”: A multiproxy, interdisciplinary and experimental approach to Paleolithic subterranean activities,” the author presents some original data combined with a synthesis of previously published work regarding the analysis of archaeological charcoals from Nerja cave and data from other caves suggested to be used for lighting purposes. The analysis of the charcoals by the author was conducted using a variety of methods which includes taxonomy and taphonomy considerations and the use of Raman spectroscopy for temperature reconstructions. A Bayesian model was used to provide a chronological framework for human occupation of caves determined by fuliginochronology. The latter two parts of the work have been conducted in collaboration with other research projects/researchers and are published in various papers referenced throughout the text.

         The original data has not been reported adequately. The earlier parts of the article read like a project summary and would benefit from being re-written in more suitable style that reflects the actual content of the paper. The original work is only concentrated in Nerja Cave, focuses on charcoals and therefore this should be the sole focus of this paper.

         The analysis of the charcoals has produced strong results at Nerja and important interpretations including the possibility of seasonal occupations and selection of Pinus for lighting within the cave. In terms of the original data the following needs to be reported in full detail including data from graphs, table and figures in excel form and full detail of codes, instrumentation and standard reference materials.

 Revisions

1. The paper should focus on Nerja Cave and specify clearly in the methodology and work packages both the original data produced and use of supporting data from other projects publications with each of their references. All figures and tables should only include data from Nerja as well.
2. Detailed reporting on instrumentation including standard reference material for each method needs to be included (unless published elsewhere – reference should be made e.g. AMS 14 data provided in...). Detail is provided in terms of the microscopes used for the charcoal identification but not for Raman for temperature.
3. All the original data needs to be provided in excel for each graph and figure and for each method e.g. charcoal and Bayesian model, which charcoals were dated and which charcoals yielded specific results. Some details are provided in tables for lux properties. Again, if the data is published elsewhere e.g., Soot layers and AMS this should be in the figure/graph legend to make it clear to the reader.
4. Full detail on the experimental methods used to reconstruct the lighting systems within the cave including full reporting of the PYTHON script (unless published elsewhere if this is the case this needs to be clearly highlighted in the text and figure ledgends).
5. Refine all the earlier parts of the paper in article format specifically the introduction section which reads like a project summary. Project partners, collaborators and funding bodies/sources should be in the acknowledgments at the end of the article.
6. Palaeolithic, P needs to be capitalised throughout the text.
7. P 4. The earliest of evidence of human occupation in caves does not relate to Neanderthals – revise to include reference to African caves e.g. Wonderwerk
8. P 5 – remove references to the Middle Palaeolithic and also to the Neolithic (soot/human occupation) The paper should focus on Upper Palaeolithic at Nerja as this is the only data discussed.
9. P10 – add a section on the GIS methodology

If any, are all the source data and materials underlying the results available?

Partly

Is the work original in terms of material and argument?

Yes

Is the work clearly and cogently presented?

Partly

Is the argument persuasive and supported by evidence?

Partly

Does the research article contribute to the cultural, historical, social understanding of the field?

Yes

Does it sufficiently engage with relevant methodologies and secondary literature on the topic?

Yes

Reviewer Expertise:

Environmental archaeology, pyroarchaeology, fire and human evolution

I confirm that I have read this submission and believe that I have an appropriate level of expertise to confirm that it is of an acceptable scientific standard, however I have significant reservations, as outlined above.

# Reviewer report: Reviewer response for version 1

**DOI:** 10.21956/openreseurope.19146.r45816

**Published:** 2024-12-14

**Authors:** Isobel Wisher

**Affiliations:** 1 Aarhus University, Aarhus, Denmark

In the paper *The “Archaeology of Light”: A multiproxy, interdisciplinary and experimental approach to Paleolithic subterranean activities*, the author presents an interdisciplinary methodological framework for analysing residues produced by Palaeolithic lighting technologies in caves. The framework integrates an impressive array of methods as part of five work packages: fieldwork in six cave sites in Spain and France; SEM-EDX and TEM-EDX analysis of charcoal samples; ethnographic comparisons; experimental replication; and statistical analysis. The results presented in the paper predominantely focuses on Nerja Cave, with some experimental results from Atxurra cave. The results present high-resolution insights into the species and morphology of wood used for fire lighting activities in Nerja cave, and a model of radiocarbon dates distinguished 12 separate phases of activity from the early Aurignacian to Copper Age, with 64 occupations identified in the later phases. There are then experimental results of lighting technologies for Axturra Cave. 

 The results presented are interesting and have important implications for understanding how studying residues from lighting technologies can illuminate insights into site use and repeated visits. However, there are some issues in the manuscript that need to be addressed. I have detailed these below, as general comments and minor points that are intended to be constructive ways the manuscript can be strengthened. In general, I think this is an interesting paper with an informative, novel approach that will contribute significantly to studying Palaeolithic lighting technologies.

 General comments:

  

1. In places, particularly before the results and discussion section, the paper reads as though text was directly integrated from the original research proposal of the postdoctoral project, for example listing the different organisations and individuals that were in the project. These kinds of details should not be in the main manuscript but the acknowledgements, and these sections should be rephrased and expanded to read more like an academic paper.
2. The third work package concerns the review of ethnographic uses of fire. As a general point, the framing of this, as it stands, is a little concerning and I urge caution about directly comparing contemporary circumpolar societies with Upper Palaeolithic people, given the problematic roots of these kinds of parallels in Palaeolithic research. I would appreciate it if the author revises this section to acknowledge that direct parallels are inappropriate, and to clarify that (I assume) she uses the ethnographic part of her research to only “broaden horizons” of possible lighting technologies that may have existed, which Western researchers are limited in understanding. Further, I think it is inappropriate to directly infer cultural behaviour from drawing ethnographic parallels. Although the author does not do this, the last sentence *“For example, there is a curious cultural relationship between the qulliq (fat lamp) and Inuit women”* concerns me for this reason. However, given that there is no discussion of the ethnogaphic uses of lighting technologies in this paper, I feel this is not needed here.
3. *“The choice of Pinus tp. Sylvestris-nigra wood for lighting-related activites seems to be a cross-cultural aspect…”* – can you expand on this to better motivate this interpretation? You mention that this species of wood is in lower percentages from the Upper Magdalenian, but it’s not clear to me a) what other suitable woody species are available throughout the Upper Palaeolithic in this region and at what percentage, b) if this species was dominant and in high percentages right up until the Upper Magdalenian, surely this means that for the majority of the Upper Palaeolithic the choice of this species of wood is directed more by resource availability, rather than cultural preference? It seems the cultural preference argument is only relevant for the Upper Magdalenian, when the presence of this species decreases.
4. I like the point regarding the use of dead wood possibly implying a seasonal component to the activity, or storage of wood resources. However, you mention that “the aerobic degradation of the wood prior to combustion could be linked to the use of dead wood”. Are there other possible explanations for the aerobic degradation, and could these be presented to better support the interpretation? Additionally, it would be nice (but not essential) to have more on the possible harvesting practices of wood. The ring curvatures imply no large logs were used – so presumably they didn’t fell entire trees, but possibly collected dead branches or harvested branches?
5. While the finding of 64 separate occupations throughout the Neolithic and Copper Age identified by analysis of micro-soot layers is really interesting, I wonder if this would be better saved for another paper? The focus of this paper is explicitly on Palaeolithic lighting systems, and this part of the paper reads as separate to this main theme.
6. The results section reads as a little disjointed in places, and it is difficult to see how each section relates to each other. The first two sections of the results are concerned with different analyses of residues from Nerja cave, but then the experimental replication section is not related to Nerja cave at all, and instead discusses Atxurra cave and replica lamps from La Mouthe (which is not one of the six case study sites presented earlier on in the manuscript). For consistency, I feel the results should explicitly focus on one case study (e.g., Nerja cave) as an example that demonstrates the efficacy of the framework and work packages presented earlier on in the paper, rather than inconsistently discussing unrelated results from different sites. If this isn’t possible, I feel there needs to be some reframing or clarification in the paper that the results are sampled from different studies as examples, but I think it would make the paper much stronger if the different approaches can be discussed for the same case study (to demonstrate how each method gave new insights into the lighting technology from one cave). 

Specific (minor) points:

  

1. The opening sentence *“The first solid data…” *would read better as *“The earliest evidence…”. *It would be more suitable to specify "in Europe" here too, given the presence of hominins using caves in South Africa that significant predate Bruniquel.
2. On “Internal Archaeological Context” – I’m not sure why this is capitalised? It may also be useful to specifically define what you mean by this.
3. *“Remarkably this this light-tool is the least frequent in the archaeological record and the one with the least light capacity”* – I feel this phrasing is a little misleading, and gives the impression that these previous studies had a research bias towards lamps over other lighting technologies. I feel it would be useful to emphasise here that stone lamps were the only clear and archaeologically visible evidence at the time to study lighting technologies, particularly given the poor resolution of cave excavations in the early 20 ^th^ Century which likely did not record charcoals. It is only recently that new excavations and developments in methods have allowed for the study of charcoal residues.
4. Ensure consistency in capitalising Palaeolithic and italicising Latin species names.
5. It is stated that there are six work packages, but only five are presented.
6. Can the radiocarbon dates be presented in the manuscript or as supplementary info, separate to figure 5? The tight overlap of the modelled dates makes it difficult to read off the ages or know the error margins of these dates.
7. Can details of the experimental design be presented in the final results section? While I know these experiments are published in more detail elsewhere, I feel a concise description of the experimental design is still needed.

If any, are all the source data and materials underlying the results available?

Partly

Is the work original in terms of material and argument?

Yes

Is the work clearly and cogently presented?

Partly

Is the argument persuasive and supported by evidence?

Yes

Does the research article contribute to the cultural, historical, social understanding of the field?

Yes

Does it sufficiently engage with relevant methodologies and secondary literature on the topic?

Yes

Reviewer Expertise:

Palaeolithic archaeology, specifically parietal and portable art and lighting related to cave art.

I confirm that I have read this submission and believe that I have an appropriate level of expertise to confirm that it is of an acceptable scientific standard, however I have significant reservations, as outlined above.

# Reviewer report: Reviewer response for version 1

**DOI:** 10.21956/openreseurope.19146.r45811

**Published:** 2024-11-29

**Authors:** Sarah Wurz, Jerome Reynard

**Affiliations:** 1 University of the Witwatersrand Johannesburg, Johannesburg, Gauteng, South Africa

**Summary:**

 This article reports results on the analysis of charcoal from Nerja Cave using various methods **, **including taxonomic identification and taphonomy. Dendroanthracological analysis of the charcoal was attempted but failed. It is suggested that *Pinus sylvestris* was selected for lighting fires within caves, in the Aurignacian to Late Magdalanian, perhaps linked to its availability in the environment. Through C14-AMS dating of 53 charcoal pieces a Bayesian model was constructed that found that Nerja Cave was visited during 12 phases in the past between around  41 000 and  3000 years ago. Through the analysis of micro-layers of soot inside a small stalagmite from Nerja Cave, it was inferred that the minimum number of 64 occupations between 7000 and 3000 years ago for the last three phases occurred.  Some experimental replication was undertaken (but not fully reported on in this article) involving wooden torches, fire-places, and replicas of sandstone lamps from La Mouthe Cave.  These sources were used to light caves with rock art in Santimamiñe, Altxerri and Atxurra and it is concluded that some of the art was placed intentionally, but the author does not make it clear where the art occurs and what the inferred intention for the placement of the art was. 

**General comments:**

 This article provides novel perspectives on the use of fire technologies used in deep caves for lighting.  The multidisciplinary approach is valuable and has much potential for further research in other regions.  The information provided in the article seems to be based on in-depth research, but the details of the research are not always provided, especially for the experimental and GIS sections. 

 The article attempts to synthesize several strands of evidence according to the following objectives (page 5):    

 1. Define and analyze the lighting systems used by Palaeolithic hunter-gatherers **in each cave**, by multiproxy and multi-analytical analysis (prioritizing on-site and non-destructive analysis). Only one cave is discussed in detail.

  2. Quantify the physical parameters and elemental characteristics, including their residual aspects, through experimental replication and monitoring. This was achieved to some extent, but not sufficient detail on the experimental replication and motivation for the materials selected and methods applied have been provided.

  3. Synthesize the data and evaluate the role of firelight in Palaeolithic hunter-gatherer societies, including its significance for the “anthropization” of caves and the performance of the first symbolic activities in them.  This has not been achieved at all.

 Insufficient data have been provided. The article makes bold claims, but this can only be substantiated if supporting data are provided.  As the paper stands the claims seem to be assertions, rather than inferences based on facts.  The data should be made available in tables and figures in the text, and as supplementary material.  Currently, this article reads as a promotion of a post-doctoral project. It should be refined to reflect the actual data discussed in the paper.

 The article needs proofreading and editing throughout: For example Palaeolithic with a capital P; consistent spelling of Palaeolithic (sometimes it is spelled Paleolithic); "charcoal" should be used for the singular and plural for,s;  ‘specially’ should be replaced with ‘especially’; It is unclear why ‘they’ and ‘we’ are used throughout when this is a single-authored publication. 

 The first two pages need to rewritten to accurately reflect what the article actually reports.

**Below are detailed comments per page:**

**Page 1: Methods**

 “Work in caves.”  Replace ‘work’ with a more suitable word.  Do not use “…..”  or “etc”.  Simply provide the facts here. The “Ethnographic review of firelight"  and "Experimental reproduction and monitoring of Palaeolithic firelight.” should not be mentioned here. 

**Page 1: Results**

 Delete “...”

 “Besides, experimental reproductions have enabled evaluation of their lighting

 potential, and provide essential information for research the visibility

 and the accessibility of Rock Art from GIS, and allow to more realistic

 virtual simulation.”  Do not start the sentence with ‘Besides’;  Divide this sentence into two.

**Page 2: Conclusions**

 Delete “In short”,

 The Conclusion on page 2 needs to be rewritten to be less hyperbolic and reflect real results.  [“In short, these data demonstrate that the *Archaeology of the Light *is **here to stay** and that it is **an essential approach** for a holistic understanding of Palaeolithic caves.”]

**Page 2: Plain Language Summary**:

 This is very general and inflated and reads more like an introduction than a summary. Much editing of this paragraph is needed.

**Page 4:**

 “The **first solid data** of “palaeospeleology”, that is, the presence of ancient humans in deep caves, were related to Neanderthals.”  There is well-established evidence that hominins have been occupying caves for well over one  million years ago, for example at Wonderwerk Cave in South Africa

 “palaeospeleology” Is this word appropriate here? This concept usually refers to the methods used to study the caves.  Archaeo-speleology refers to the study of the use of caves in the Palaeolithic past

 Internal Archaeological Context – define this

 “In my Ph.D., I studied……. project is still ongoing in the framework of a postdoctoral contract MSCA-PF (2022–2024).”  Is this information needed here? It breaks the flow of the academic discussion.  Perhaps it could move to an acknowledgement/funding section at the end.

 Figure descriptions should be in captions, not the text, as for Figure 1a-c.

*“ *figure 1b scattered charcoals linked to the use of wooden torches from Alkerdi 2 cave”  No other data from Alkerdi 2 are discussed in the paper. A figure of charcoal from Nerja cave thus needs to be provided. 

 How does the author know these charcoal scatters are from wooden torches?  Please justify.  Such scatters are routinely found in cave sites dating back well into the Pleistocene, where there is no evidence or hypotheses for the use of torches.  

**Page 5:  **“This research focuses on outstanding remains of palaeolithic lighting from **six European caves**, one of which has been declared a World Heritage Site by UNESCO (Altxerri cave) (Figure 2)”  . This creates the impression that data from all six caves will be discussed in the article, in reality only Nerja Cave is discussed.

 “Specifically, the inner anthropization of the selected sites integrates different phases of the Upper Palaeolithic (35–12 ka. BP), including a period of the Middle Palaeolithic (176 ka. BP).”  Only Upper Palaeolithic evidence is discussed in this paper, it is not clear why the Middle Palaeolithic is included in this sentence. 

 “3. Ethnographic review of the firelight”.   There is no ethnographic review in this paper??

 Hominin not humanoid

**Page 6**:

 “For example, there is a curious cultural relationship between the qulliq (fat lamp) and Inuit women (Billson & Mancini, 2007).”  Replace ‘curious’ with a more appropriate word such as thought-provoking.

 “ *Pinus *tp. *sylvestris*- *nigra *is the most common anthracological type found in the interior of caves and is linked to artificial lighting in the Upper Palaeolithic, specifically in the pre-Magdalenian period.”  Is this true for all Upper Palaeolithic caves everywhere, also from different environments?  Qualify this statement by referring to specific regions/countries and their environments.

**Page 7:**

 “Also, thanks to our results, we can suggest that the choice of *Pinus *tp. *sylvestris*- *nigra *wood for lighting-related activities seems to be **a cross-cultural aspect, shared by palaeogroups over a long period of time (30,000 years)”.** This is a very strong inflated statement that needs more evidence.

 “Concerning the taphonomic alterations of the analysed charcoals, we observed different anomalies linked to the combustion process, such as vitrification (54.14%) and shrinkage cracks (32.41%).” Provide a table for the taphonomic data – this is essential given the importance attached to taphonomy in this paper.

**Page 8:**

 “Through the C14-AMS dating of 53 of these charcoals, we constructed a high-precision Bayesian…”  Be specific. Which charcoal from where, 53 out of how many charcoal pieces?

 “The Bayesian model suggests at least **12 distinct phases **of visits to the interior of Nerja cave between **41,218 and 3299 cal BP”.  **Provide a table for these data.

**Page 9**

 Figure 5 is very interesting! Unfortunately, one cannot see the details within the figure.

 “Through the identification, microcounting and dating of micro-layers of soot …..present inside a small stalagmite”  Explain exactly where the stalagmite was found to provide evidence that this might be directly related to visits to the cave.  Another speleothem fragment is also mentioned in the article (page 9), but it is also not explained where it was found. More information on these structures is needed (photographs and a map indicating their positions)

**Page 10**

 “The **experimental replication of Palaeolithic light **was monitored in an endokarst …”– there is no information on the experimental design- this is crucial information that cannot be left out!

 “Birch bark was included among the juniper branches as a kind of tinder to start combustion. Similarly, the wood was broken up to facilitate combustion (through easier oxygenation) and impregnation with non-ligneous fuel. Thus, pine resin, animal fat, or a combination were added in some cases to assess the function of the torches with those fuel types”  Why were these elements added -  a motivation is needed.

 Fireplaces: A motivation should be provided for  why the fireplaces were constructed in this way

 Lamps: Provide image(s) of the experimental lamps. "We developed pioneer methodologies in GIS environments for the quantitative determination of the degree of visibility, gauging and difficulty of access to Palaeolithic Art”. 

 There should be a new section on the rock art and GIS.  

 No information on the art is provided, and the GIS methodology is not explained.

**Page 11:**

 “Showing that the panel is located over a cornice …” Figure 9 does not indicate the position of the rock art.  Where is the cornice?

**Page 12:**

 Rock art imagery is referred to, but no information is provided.

 “As a preliminary conclusion, the results obtained seem to show that the artists’ choice of panel was directly affected by the difficulty of transit.”  Explain what is meant here – what is meant by "difficulty of transit"?

If any, are all the source data and materials underlying the results available?

Partly

Is the work original in terms of material and argument?

Yes

Is the work clearly and cogently presented?

Partly

Is the argument persuasive and supported by evidence?

Partly

Does the research article contribute to the cultural, historical, social understanding of the field?

Yes

Does it sufficiently engage with relevant methodologies and secondary literature on the topic?

Partly

Reviewer Expertise:

Palaeolithic archaeology

We confirm that we have read this submission and believe that we have an appropriate level of expertise to confirm that it is of an acceptable scientific standard, however we have significant reservations, as outlined above.
